# High-quality reference genome sequences of two coconut cultivars provide insights into evolution of monocot chromosomes and differentiation of fiber content and plant height

**DOI:** 10.1186/s13059-021-02522-9

**Published:** 2021-11-04

**Authors:** Shouchuang Wang, Yong Xiao, Zhi-Wei Zhou, Jiaqing Yuan, Hao Guo, Zhuang Yang, Jun Yang, Pengchuan Sun, Lisong Sun, Yuan Deng, Wen-Zhao Xie, Jia-Ming Song, Muhammad Tahir ul Qamar, Wei Xia, Rui Liu, Shufang Gong, Yong Wang, Fuyou Wang, Xianqing Liu, Alisdair R. Fernie, Xiyin Wang, Haikuo Fan, Ling-Ling Chen, Jie Luo

**Affiliations:** 1 Hainan Key Laboratory of Tropical Oil Crops Biology, Coconut Research Institute, Chinese Academy of Tropical Agricultural Sciences, Wenchang, China; 2 College of Tropical Crops, Hainan University, Haikou, 570228 China; 3 Sanya Nanfan Research Institute of Hainan University, Hainan Yazhou Bay Seed Laboratory, Sanya, 572025 China; 4 Sanya Research Institute of Chinese Academy of Tropical Agricultural Sciences, Sanya, China; 5 National Key Laboratory of Crop Genetic Improvement, Huazhong Agricultural University, Wuhan, 430070 China; 6 State Key Laboratory for Conservation and Utilization of Subtropical Agro-bioresources, College of Life Science and Technology, Guangxi University, Nanning, 530004 China; 7 College of Life Sciences, Shaanxi Normal University, Xi’an, 710119 China; 8 Center for Genomics and Computational Biology, North China University of Science and Technology, Tangshan, China; 9 Max Planck Institute of Molecular Plant Physiology, 14476 Potsdam-Golm, Germany

## Abstract

**Background:**

Coconut is an important tropical oil and fruit crop whose evolutionary position renders it a fantastic species for the investigation of the evolution of monocot chromosomes and the subsequent differentiation of ancient plants.

**Results:**

Here, we report the assembly and annotation of reference-grade genomes of *Cn. tall* and *Cn. dwarf*, whose genome sizes are 2.40 Gb and 2.39 Gb, respectively. The comparative analysis reveals that the two coconut subspecies diverge about 2–8 Mya while the conserved Arecaceae-specific whole-genome duplication (ω WGD) occurs approximately 47–53 Mya. It additionally allows us to reconstruct the ancestral karyotypes of the ten ancient monocot chromosomes and the evolutionary trajectories of the 16 modern coconut chromosomes. Fiber synthesis genes in *Cn. tall*, related to lignin and cellulose synthesis, are found at a higher copy number and expression level than dwarf coconuts. Integrated multi-omics analysis reveals that the difference in coconut plant height is the result of altered gibberellin metabolism, with both the GA20ox copy number and a single-nucleotide change in the promoter together leading to the difference in plant height between *Cn. tall* and *Cn. dwarf*.

**Conclusion:**

We provide high-quality coconut genomes and reveal the genetic basis of trait differences between two coconuts through multi-omics analysis. We also reveal that the selection of plant height has been targeted for the same gene for millions of years, not only in natural selection of ancient plant as illustrated in coconut, but also for artificial selection in cultivated crops such as rice and maize.

**Supplementary Information:**

The online version contains supplementary material available at 10.1186/s13059-021-02522-9.

## Background

Coconut (*Cocos nucifera*, 2*n =* 32), a member of the monocotyledonous Arecaceae (Palmaceae) family and Arecoideae sub-family. It is an important tropical oil and fruit crop, widely distributed across 93 tropical countries [1, 2]. In 2018, the global coconut acreage was 12.2 million hectares and the total production was 61.8 million tons (United Nations Food and Agriculture Organization (FAO) statistics; see URLs). As a portable source of food and water, coconuts have played a key role in human navigation, establishing trade routes, and the ability to settle on land in the Pacific Rim and regions throughout the Old World tropics [3, 4]. Given its various uses as a source of healthy drink, fiber, building materials, charcoal, and oil (for cooking, medicinal usage, industrial applications, and biofuels production), coconut is widely acknowledged as a tree of life [5, 6]. Indeed, the history of the spread and cultivation of this species is fundamentally intertwined with human history in the tropics [7, 8]. Coconuts are divided into two subgroups based on their independent evolutionary origin: one from the indo-Atlantic Ocean population and the other from the Pacific Ocean subgroup [9, 10], the latter is the most likely center origin of the coconut [11, 12]. The oldest fossil record available is from India suggesting that the origin of coconut is approximately 66 million years ago (Mya). This observation places the origin of the coconut at the end of Cretaceous or between the end-Mesozoic and early Tertiary era, indicating that coconut is an ancient angiosperm crop [1]. Since the angiosperms are proposed to have arisen in the Cretaceous period, the evolutionary position of coconut thus renders it a fantastic species for investigating the evolution of monocot chromosomes and the differentiation of ancient plants.

Coconuts can be divided into two distinct categories according to their morphological characteristics and breeding habits: *Cocos nucifera* tall (*Cn. tall*) and *Cocos nucifera* dwarf (*Cn. dwarf*) (Additional file 1: Figure S1a). *Cn. tall* is hardy, is cross-pollinating, and has a medium to large nut size [13, 14], it can adapt to a wide range of environment conditions, and grow to a height of approximately 20–30 m yet being slow to mature (flowering 8–10 years after planting). By contrast, *Cn. dwarf* is early-flowering (4–6 years after planting), self-pollinating, can grow to a height of 10–15 m and produces a number of small nuts [3, 15, 16]. Dissection of the genetic variation causing the difference between *Cn. tall* and *Cn. dwarf* is essential for the development of improved varieties with resistance to diseases, pests, and climate change and for refining oil yield. In the early 20th century, conventional breeding schemes were carried out to improve coconut yield and quality characteristics [17, 18]. More recently, molecular breeding techniques can allow marker-assisted selection and genotyping in coconut germplasm and speed up the laborious and time-consuming process of conventional breeding in this long-lived tree species [19, 20]. However, these aspects in coconut are still under-studied due to the lack of high-quality reference genome.

Here, we report the de novo assembly of two representative coconut individuals belonging to *Cn. tall* and *Cn. dwarf* type by using Nanopore single-molecule sequencing and Hi-C technology. We predict and annotate 29,897 and 28,111 protein-coding genes in the *Cn. tall* and *Cn. dwarf* genome, respectively, the independently assembled contig N50s of which were 2.93 and 14.29 Mb, respectively. The high-quality coconut genomes and their key positioning in the evolution of monocots allowed us to reconstruct an ancestral karyotype with 10 proto-chromosomes shared by other monocot plants in order to understand its evolution history. Comparative analysis of the *Cn. tall* and *Cn. dwarf* genome revealed that the two coconut ecotypes diverged about 2–8 Mya while the conserved Arecaceae-specific whole-genome duplication (ω WGD) event occurred approximately 47–53 Mya. In addition, this study provided insights into fiber content, salt tolerance, plant height, and fatty acid content. Multi-omics-based genome-wide association analysis and the detection of gene family expansion revealed that the gibberellin (GA) biosynthetic enzymes GA-20 oxidase (GA20ox) played a crucial role in the divergence of tall and dwarf heights traits in coconut.

## Results

### Genome assembly and annotation

**Fig. 1:**
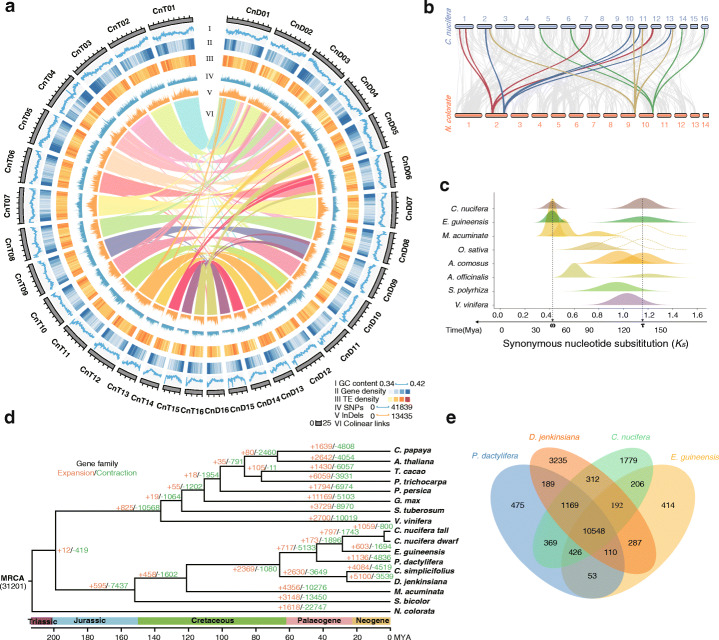
*Cocos nucifera* genome assembly, and genomic features. **a** The circos diagram of *Cn. tall* and *Cn. dwarf*. The circles from outer to inner separately represented GC (guanine-cytosine) content (I); gene density (II); transposable element (TE) density (III); SNPs density (IV); InDels density (V); Colinear links (VI). **b** Genome collinear analysis between *C. nucifera* and *N. colorata* genomes (window size of 1 Mb). **c** Density distributions of the *Ks* values for homologous genes. **d** Phylogenetic tree of *Cn. tall*, *Cn. dwarf*, and other 15 angiosperm species including their divergence time based on orthologues of the single-gene family. The circles represent the diploid events and the squares represent the triploid events. **e** Clusters of orthologous and paralogous gene families in *C. nucifera*, and three palm species

We used a multifaceted sequencing approach for chromosome-scale assembly. For the *Cn. tall* sample, 227 Gb of Nanopore sequencing reads (~114×), 124 Gb of Illumina reads for genome correction, and 213 Gb of high-throughput chromosome conformation capture (Hi-C) reads were generated and applied to genome assembly (Additional file 2: Table S1). For *Cn. dwarf*, a corresponding set of 249 Gb Nanopore sequencing reads (~102×), 126 Gb of Illumina reads and 270 Gb of Hi-C reads for the *Cn. dwarf* were obtained (Additional file 2: Table S1). We achieved a de novo assembly of 2.40 Gb for the *Cn. tall* genome and 2.39 Gb for the *Cn. dwarf* genome which are very close to the estimated genome size of ~ 2.42 Gb and ~ 2.44 Gb using *k*-mer distribution analysis (Additional file 1: Figure S1b). The assemblies had contig N50s of 2.9 Mb and 14.3 Mb, respectively (Table 1). We next applied Hi-C data to order and orient the resulting contigs and thereby allow chromosome-level genome assemblies, among which 99.33% of the *Cn. tall* and 99.13% of the *Cn. dwarf* reads were anchored to 16 (2*n =* 32) chromosomes (Fig. 1a, Additional file 1: Figure S2-4, Additional file 3: Table S2 and Additional file 4: Table S3). To assess the genome accuracy and completeness of *Cn. tall* and *Cn. dwarf*, raw Illumina paired-end reads were mapped to the assembled genomes with the mapping rate of 99.59% and 99.80%, respectively. Furthermore, raw RNA-seq reads from multiple tissues were mapped to the assembled genomes with a total of over 94% of these mapping across each assembly (Additional file 5: Table S4 and Additional file 6: Table S5). Benchmarking Universal Single-Copy Orthologs (BUSCO) analysis indicated that 97.1% and 97.4% conserved single-copy eukaryotic genes were captured in *Cn. tall* and *Cn. dwarf* genomes, respectively (Additional file 7: Table S6).

**Table 1:** Summary of genome assembly and annotation

	***Cn. tall***	***Cn. dwarf***
Number of contigs	2433	401
Total size of contigs (bp)	2,393,894,742	2,399,649,694
Longest contig	34,251,876	56,639,188
Contig N50 count	196	50
Contig N50 length (bp)	2,927,039	14,296,645
Contig N90 count	1044	177
Contig N90 length (bp)	442,494	3,844,418
GC content	37.49	37.57
Number of protein-coding genes	29,897	28,111
Mean transcript length (bp)	1698	1709
Mean exon length (bp)	272	272
Mean exon per mRNA	6.23	6.27
Total repetitive sequences size (% of genome)	2,000,576,416 (83.55%)	2,015,579,366 (84.00%)

We subsequently annotated repetitive elements, non-coding RNAs, and protein-coding genes for the two genome assemblies (Table 1). The two genomes contained 83.61% (*Cn. tall*) and 83.83% (*Cn. dwarf*) transposable elements (TEs), of which retrotransposons accounted for 72.20% and 73.65% of the respective genomes (Fig. 1a, Additional file 8: Table S7). Long terminal repeats (LTRs) formed the most abundant category of TEs, with LTR/Copia and LTR/Gypsy occupying 48.80% and 19.79% of *Cn. tall* genome and 51.54% and 18.54% of *Cn. dwarf* genome (Additional file 1: Figure S5a and b, Additional file 8: Table S7). Lengths of the majority of TEs were in the range of 100–1000 bp (Additional file 1: Figure S5c). Estimation of LTR insertion time revealed that the burst of retrotransposon multiplication in *Cn. tall*, *Cn. dwarf*, and *Elaeis guineensis* [21] had happened about 3 Mya, earlier than in *Phoenix dactylifera* [22] (Additional file 1: Figure S5e).

A total of 29,897 and 28,111 protein-coding genes were predicted for *Cn. tall* and *Cn. dwarf* with the mean coding lengths of 1698 and 1709 bp, respectively (Table 1), while 81.1% (*Cn. tall*) and 83.4% (*Cn. dwarf*) of protein-coding genes were supported by the Illumina RNA-seq reads and the single-molecule long-read transcriptome data. About 96.4% and 96.3% of these genes in *Cn. tall* and *Cn. dwarf* had significant functional annotation by matching to Clusters of Orthologous Groups of proteins (COGs), Kyoto Encyclopedia of Genes and Genomes (KEGG), Non-redundant Protein Sequence (NR), SwissProt, and Gene Ontology (GO) databases, respectively (Additional file 9: Table S8). In addition, we identified 5795 and 7409 non-coding RNAs containing ribosomal RNAs, transfer RNAs, microRNAs, and small nuclear RNAs (Additional file 10: Table S9).

### Genome duplication and evolution

Compared with the genome of *Nymphaea colorata* [23], the most basal lineage of angiosperms, there were 1563 collinear segments, and 28.7% (6907/24,059) of *N. colorata* genes showed high similarity with a set of four homologous segments in the *C. nucifera* genome, and 50.4% (15,057/29,897) genes in *C. nucifera* possessed high collinearity with two homologous segments in *N. colorata* (Fig. 1b, Additional file 1: Figure S6a).

We then investigated whether the WGDs identified in the coconut genome reflected the same or different evolutionary events reported in related plant genomes (Additional file 11: Table S10 and Additional file 12: Table S11), by resolving the orders and dates of WGDs alongside the split of coconut and oil palm. For this purpose, we extracted colinear paralogous pairs from their respective WGDs, in each genome of coconut and six representative monocots *E. guineensis*, *Musa acuminate* [24], *Oryza sativa* [25], *Ananas comosus* [26], *Asparagus officinalis* [27], *Spirodela polyrhiza* [28, 29], and one eudicot genome *Vitis vinifera* [30], and orthologous pairs were also identified among these plants. By characterizing *Ks* values between paralogs, and those between orthologs, *C. nucifera* and *E. guineensis* had a recent common WGD with the exception of an ancient polyploidization (Fig. 1c, Additional file 1: Figure S6b and c, Additional file 11: Table S10 and Additional file 12: Table S11).

We performed a correction procedure to *Ks* values before evolutionary dating, by exploiting the facts that the studied monocots had diverged from the eudicot representative, *V. vinifera*, at the same time. After evolutionary correction, by assuming the monocot-eudicot divergence at 163–184 Mya, we dated the other evolutionary events (Fig. 1c, Additional file 1: Figure S6d). Based on this study, we found that the divergence of *C. nucifera* and *E. guineensis* occurred about 17–19 Mya, their shared *ω* WGD occurred about 47–53 Mya, and the *τ* WGD occurred 129–146 Mya. Another four Arecaceae species recently released with their genome sequences were compared with coconut. An integration of shared gene collinearity and *Ks* distribution showed that all these compared Arecaceae went through the two rounds of WGDs *τ* and *ω* (Additional file 1: Figure S7 and 8). Evolutionary dating based on *Ks* showed similar occurrence times as described above (Additional file 1: Figure S9, Additional file 13: Table S12).

To further date evolutionary events, we compared the single-copy protein-coding genes in *C. nucifera* with their orthologs in *E. guineensis*, *P. dactylifera*, *Calamus simplicifollus*, *Daemonorops jenkinsiana*, *M. acuminata*, *Sorghum bicolor*, *Arabidopsis thaliana*, *Carica papaya*, *Theobroma cacao*, *Populus trichocarpa*, *Glycine max*, *Pyunus persica*, *V. vinifera*, *Solanum tuberosum*, and *Allium tuberosum*. These data were used to construct a time-calibrated phylogenetic tree (Fig. 1d, Additional file 14: Table S13). Phylogenetic analysis showed that *C. nucifera* had the highest similarity with *E. guineensis*. Meanwhile, the divergence time between *Cn. tall* and *Cn. dwarf* was dated to between 2 and 8 Mya. Based on both the phylogenetic tree and the above *Ks* inference, the *τ* WGD was detected to be shared by all species of the core monocotyledon lineages.

Compared with the most recent common ancestor of coconut, oil palm, and date palm, gene family expansion was 6.75% (1019/15,103) in *Cn. tall*, and 3.46% (508/14689) in *Cn. dwarf*, and the gene family contraction was 10.7% (1574) in *Cn. tall* and 4.9% (740/14,689) in *Cn. dwarf* (Fig. 1d). Enrichment analysis of the expansion genes had clustered to 50 GO categories, among which the highest *P* (1.75 e−14) value in *Cn. tall* was “regulation of cell growth” (Additional file 1: Figure S10a). The contracted genes were enriched into 15 GO categories with the most enriched category being “plastid outer membrane” (Additional file 1: Figure S10b). While comparing gene families in *C. nucifera* with other three Arecaceae species including *E. guineensis*, *P. dactylifera*, and *D. jenkinsiana*, we found that 70.32% (10,548/15,001) of the gene families in *C. nucifera* were shared among all four genomes, and only 11.86% (1779/15,001) of gene families were *C. nucifera* specific (Fig. 1e). GO enrichment analysis of these specific gene families revealed 31 significantly enriched GO categories, mainly concentrated in biological process category, including “vitamin metabolic process,” “terpenoid biosynthetic process,” and “alpha-amino acid biosynthetic process” (Additional file 1: Figure S11a and b).

### Ancestral karyotype of monocots and coconut karyotype evolution

**Fig. 2:**
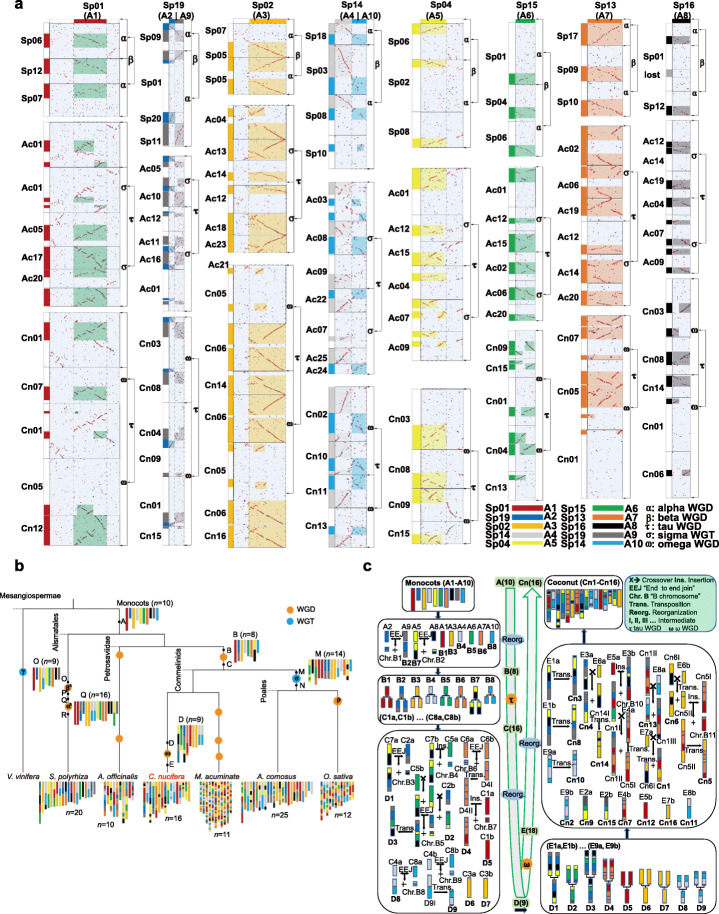
Inference of monocot proto-chromosomes and reconstruction of evolutionary trajectories of the extant coconut (Cn) chromosomes. **a** Identification of proto-chromosomes through evaluating shared homology between extant chromosomes. The chromosomes of the outgroup monocot, *S. polyrhiza* (Sp), were compared to those in the other monocots. Inferred proto-chromosomes, A1–A10, were each shown in a specific color. Dotplots show shared gene collinearity, and orthologous correspondence were shown by filled and transparent rectangles. Greek letters were used to show which event produced the chromosomes with arrows pointed to. Cn: *C. nucifera*; Ac: *A. comosus.* **b** Plant phylogeny and reconstructed monocot proto-karyotypes. Proto-chromosomes at specific evolutionary nodes were inferred, such as nine proto-chromosomes before the WGD omega. Chromosomes are denoted with color scheme showing 10 monocot proto-chromosomes. WGD and WGT are shown, and some of which are named with Greek letters. **c** Reconstruction of evolutionary trajectories from monocot proto-chromosomes to form the extant coconut (Cn) chromosomes. Proto-chromosomes on different evolutionary nodes (A–E) were reconstructed and correspondingly named. Greek letters were used to relate to each polyploidization event. “EEJ” indicates end-to-end joining of chromosomes, “Reorg.” indicates reorganization, and “Ins.” indicates insertion. The sign of “Chr. B” represents mini-chromosome (B chromosome) produced by the “EEJ” process

The high-quality coconut genome and its key positioning within the evolution of monocots render it possible to reconstruct the ancestral karyotype of monocots. To reconstruct the ancestral karyotype of monocots, the same seven representative and well-assembled genomes listed in Fig. 2b were used for comparison with *C. nucifera*. By inferring intergenomic gene collinearity, we mapped the above genomes onto *C. nucifera*, and the determined ratios of the best-matched homologous regions between *C. nucifera* and *E. guineensis*, *A. comosus*, *A. officinalis*, and *S. polyrhiza* were 2:2, 2:3, 4:4, and 4:4, respectively, revealing how many times each of them was affected by WGDs (Additional file 1: Figure S12 and 13a, Additional file 15: Table S14). Through exploring the gene collinearity (Fig. 2a, Additional file 1: Figure S13a-c), we constructed an ancestral karyotype with 10 proto-chromosomes shared by monocot plants of the other phylogenetic nodes (Fig. 2b). Having obtained the reconstructed ancestral proto-chromosomes, at the meantime, we deduced the most possible evolutionary trajectories to form the extant chromosomes of *C. nucifera*, *S. polyrhiza*, and *A. comosus* (Fig. 2c, Additional file 1: Figure S14 and 15), and reconstructed the ancestral chromosomes of the other three species *O. sativa*, *M. acuminate*, and *A. officinalis* (Fig. 2b, Additional file 1: Figure S13d-f, Additional file 16: Note S1).

By comparing the basal monocot *S. polyrhiza* with *C. nucifera* and *A. comosus* genomes, we deduced 10 proto-chromosomes (Fig. 2a) before the divergence of the studied monocots (node A) (Fig. 2b). A close check of shared collinearity between extant plant chromosomes helped to identify that certain extant chromosomes arose from parts of the other chromosomes of other plants thereby revealing their antiquity. For example, chromosome Ac17, its *τ* WGD, and their ortholog Cn12 share homology in their full length. Ignoring certain segmental inversions or minor deletions, we deduced that they have preserved the structure of their common ancestral proto-chromosome (Fig. 2a), which we hereafter refer to as A1. Proto-chromosome A1 has homologous regions in the other extant chromosomes, such as Ac20 and Cn01, which were surely produced by fusing with the other ancient chromosomes. The proto-chromosome has split or complement homology with Cn01 and Cn05, which can be explained if two ancient chromosomes exchanged arms or DNA with one another. In a similar manner, we inferred the other nine monocot proto-chromosomes A2–A10, and proto-chromosomes of the other evolutionary nodes studied (Fig. 2b). In doing so, we revealed that there were eight proto-chromosomes before the *τ* WGD, but before the recent *ω* WGD, the ancestor of *C. nucifera* and *E. guineensis* had 9 proto-chromosomes (Fig. 2b).

After deducing the proto-chromosomes, we inferred the likely evolutionary trajectories required to form the extant chromosomes (Fig. 2c). Through two independent end-end join (EEJ), the monocot 10 proto-chromosomes were reduced to eight chromosomes before the *τ* WGD (node B), with A2 and A9 joining to produce B2, and A5 and A8 joining to produce B7, respectively. The other monocot proto-chromosomes were preserved within genomes of node B. After *τ* polyploidization, the ancestral genome at node B doubled into 16 within genomes of node C (Fig. 2c). By comparing to *S. polyrhiza* and *A. comosus*, we deduced the ancestral genome of extant *C. nucifera* within node D. A relatively complex reorganization involving five EEJ, two arm-exchanging crossovers, and two nested chromosome fusions led to a chromosome number of nine. The recent *ω* WGD doubled the chromosome number to 18 at node E. After five crossovers to exchange DNA and two nested chromosome fusions, eventually, formed the extant 16 chromosomes of *C. nucifera* (Fig. 2c, Additional file 16: Note S1).

### Comparative genome analysis between *Cn. tall* and *Cn. dwarf*

**Fig. 3:**
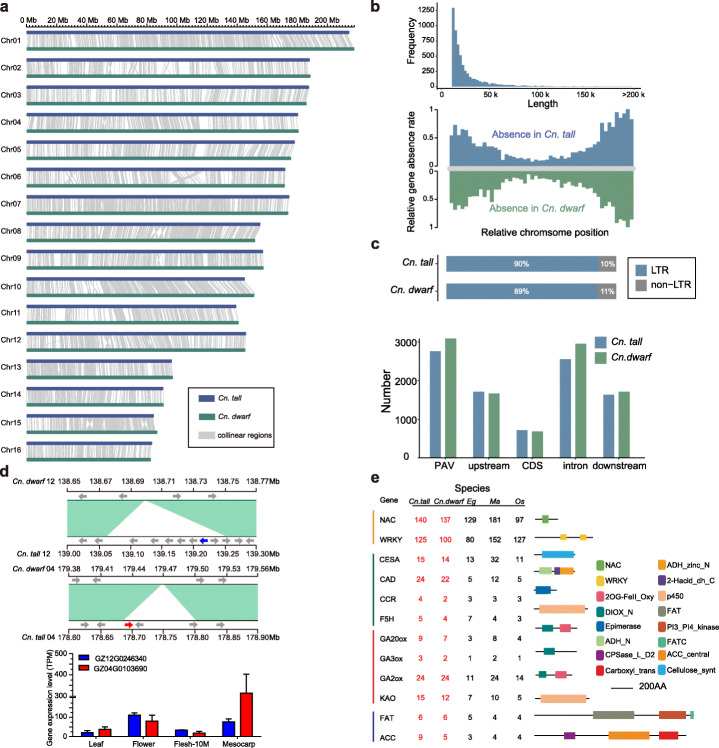
Comparative genomic analysis between *Cn. tall* and *Cn. dwarf*. **a** Genome collinearity between *Cn. tall* and *Cn. dwarf*. The size less than 100 kb of colinear syntenic blocks are filtered out. **b** PAV distribution across the genomes of *Cn. tall* and *Cn. dwarf*. **c** The upper graph shows the percentage of PAVs with TEs in *Cn. tall* and *Cn. dwarf*. the other graph shows the distribution of PAVs in different gene parts. **d** Sequence comparison between *Cn. tall* genome and *Cn. dwarf* genome at chromosome 04 and 12, the figure below shows the expression of genes special presence in *Cn. tall* in different tissues of coconut. The values on the *y*-axis correspond to the expression levels of cellulose synthase A (*GZ04G0103690*) and gibberellin-regulated protein (*GZ12G0246340*). Cellulose synthase A, gibberellin-regulated protein, and other genes are indicated with blue, red, and grey, respectively. **e** Gene copy number and domain architecture of genes, which related to differences in *Cn. tall* (*Cn. T*), *Cn. dwarf* (*Cn. D*), *E. guineensis* (*Eg*), *M. acuminate* (*Ma*), and *O. sativa* (*Os*). The red numbers indicate differences in the number of genes between coconuts and other species

The genome sequence alignment between *Cn. tall* and *Cn. dwarf* reveals high collinearity (Fig. 3a, Additional file 1: Figure S16a, Additional file 17: Table S15 and Additional file 18: Table S16), and a total of 22,918,475 SNPs and 12,530,050 indels was identified between *Cn. tall* and *Cn. dwarf*. Genomic variant annotations and functional effect prediction showed 2264 genes were highly impacted by these SNPs/InDels (Additional file 1: Figure S16b, Additional file 19: Table S17). Moreover, characterization of presence/absence variations (PAVs) showed there were 69,012 discrepant segments between the two coconut accessions with a length of 297.98 Mb (Fig. 3a, Additional file 1: Figure S16c), among which 37,672 and 31,340 segments were absent in *Cn. tall* and *Cn. dwarf*, respectively (Fig. 3b, Additional file 20: Table S18). There were 2889 genes in *Cn. tall* and 3215 genes in *Cn. dwarf* in these PAV regions. Enrichment analysis of genes absent in *Cn. dwarf* showed that they were significantly (− log_10_^*p*^ > 10) enriched in acyl-binding function, which plays a crucial role in the biosynthesis of fatty acids. These results were in accordance with lower oil content in *Cn. dwarf* (Additional file 1: Figure S13b). Interestingly, 90% of PAVs were related to TEs, which indicated that the majority of PAVs between *Cn. tall* and *Cn. dwarf* may be associated with the activation or structural variation of TEs (Fig. 3c). Characterization of PAVs of TEs in the two coconut accessions indicated that the length frequency distribution peaked at approximate 10 kb. The variable TEs were comprised of LTR/Gypsy (12%), LTR/Copia (13%), LTR (33%), and others (42%) (Additional file 1: Figure S5d).

In addition to PAVs, twenty-one large structure variations (SVs) (> 1 Mb), including inversions and translocations, were identified between *Cn. tall* and *Cn. dwarf* genomes, with their lengths varying from 1,130,470 to 21,473,489 bp (Additional file 1: Figure S16d, Additional file 21: Table S19). Some SVs may be associated with the divergence of the agronomic traits between *Cn. tall* and *Cn. dwarf*. For example, a large segment insertion (0.18 Mb) on the chromosome 12 of the *Cn. tall* genome containing eight genes, one of which was annotated as gibberellin-regulated protein gene (*GZ12G0246340*) and had higher expression levels in coconut flower and mesocarp than in other tissues, which may have an important impact on coconut plant height traits. Another example involves in a deletion (0.12 Mb) on chromosome 4 of *Cn. dwarf* resulting in the loss of a cellulose synthase A gene (*GZ04G0103690*) associated with cellulose biosynthesis, which had very high expression level in coconut mesocarp consisted of cellulose (Fig. 3d).

The protein-coding genes in *Cn. dwarf* and *Cn. tall* were classified into 14,200 and 14,631 gene families, respectively (Additional file 1: Figure S11c). By comparing the gene family annotation between *Cn. tall* and *Cn. dwarf*, there were 13,829 common gene families; meanwhile, 758 were specific to *Cn. tall* and 370 were specific to *Cn. dwarf*. Among the *Cn. tall*-specific gene families, GO and KEGG enrichment analysis mainly involved “response to gibberellin,” “gibberellic acid mediated signaling pathway,” “auxin biosynthetic process,” “regulation of seed germination,” and other related items (Additional file 1: Figure S17a and b). Among the *Cn. dwarf* -specific gene families, GO and KEGG terms were mainly enriched in “glycolipid biosynthetic process,” “oligosaccharide biosynthetic process,” and other related items (Additional file 1: Figure S17c and d). We conducted a comprehensive analysis of the genes related to their different traits and compared them with the corresponding homologous genes in oil palm and rice (Fig. 3e). *NAC* and *WRKY*, families of transcription factors that have been studied extensively for resistance to salt stress in plants, have 11 and 125 members in *Cn. tall*, respectively, which are more than the other three species including *Cn. dwarf*. The copy number of many key genes in the gibberellic acid (GA) synthesis pathway that affects plant height, including GA20ox, GA-3 oxidase (GA3ox), and *ent*-kaurenoic acid oxidase (KAO), was more numerous in *Cn. tall* than *Cn. dwarf*. Similarly, genes related to lignin and lipid synthesis were more numerous in *Cn. tall*. These results reveal that the expansion of the abovementioned genes in *Cn. tall* has caused differences in traits such as salt tolerance, fiber content, plant height, and fatty acid content in *Cn. tall* and *Cn. dwarf*.

### Genomic insights on fiber formation and regulation in coconut

**Fig. 4:**
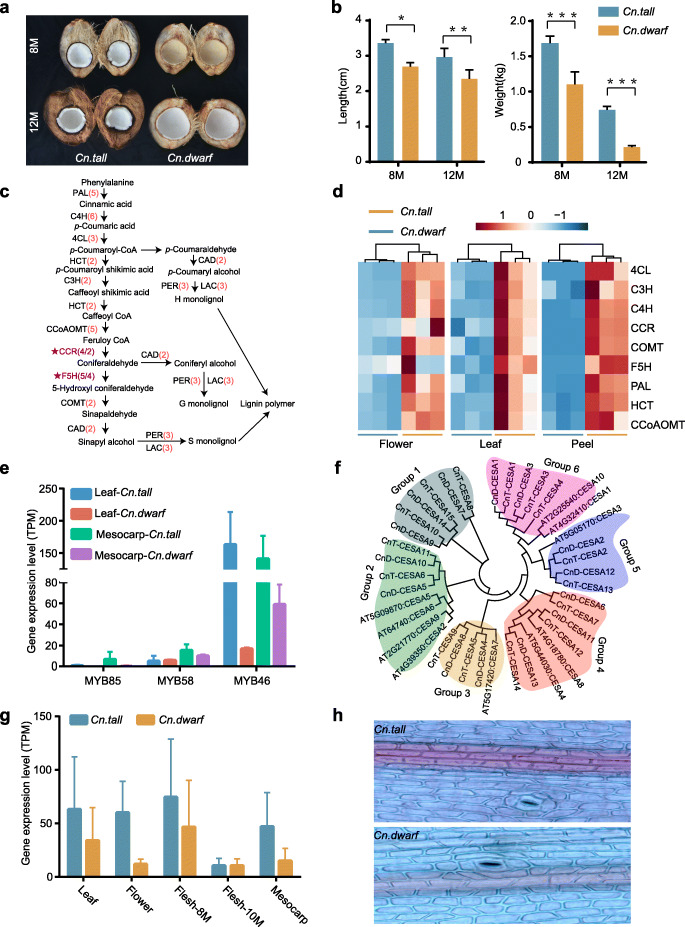
Molecular mechanism of fiber content difference between *Cn. tall* and *Cn. dwarf*. **a** Fruits of *Cn. tall* and *Cn. dwarf* with a growth period of 8 months and 12 months. **b** The average thickness and weight of coconut fiber in the growth period of 8 months and 12 months. **c** Synthetic pathway of coconut lignin. The red numbers indicate the same number of these genes in *Cn. tall* and *Cn. dwarf*, while the purple numbers indicate different numbers. **d, e** The expression patterns of structural genes (**d**) and regulatory genes (**e**) related to lignin metabolism and synthesis in different tissues of *Cn. tall* and *Cn. dwarf*. **f** The phylogenetic tree analysis of the key gene family CESA for coconut cellulose synthesis. **g** The expression pattern of CESA gene related to cellulose synthesis in different tissues of coconut. **h** Epidermal cells of leaves of *Cn. tall* and *Cn. dwarf* stained by phloroglucinol/HCl

The rich fiber content of coconuts allows coconuts to drift at sea, which is an important factor in the spread of coconuts across oceans and thereby the globe. In order to study the difference in coconut fiber content between *Cn. tall* and *Cn. dwarf*, we collected coconuts that were developed for 8 and 12 months for comparative analysis (Fig. 4a). The size of *Cn. tall* fruits during the same growth period was significantly larger than that of *Cn. dwarf*, and the thickness and weight of fiber in *Cn. tall* were significantly higher than those of *Cn. dwarf* (Fig. 4b). The above results confirm that there is significant difference in fiber content between *Cn. tall* and *Cn. dwarf*. Compared with *Cn. dwarf*, *Cn. tall* is rich in fiber which provides convenient conditions for coconuts to drift and spread at sea. For exploring the molecular mechanism of the metabolic synthesis and regulation of coconut fiber, we identified the key genes in the metabolic synthesis pathway of the two main components of coconut fiber, lignin, and cellulose. In the phenylpropanoid metabolic pathway related to lignin synthesis, a total of 117 key genes for lignin synthesis were identified. Among them, the number of CCR and F5H genes in the *Cn. tall* was significantly higher than that in *Cn. dwarf* (Fig. 4c). We conducted transcriptome analysis on the coconut mesocarp grown for 8 months and 10 months, with the results revealing that the gene expression of lignin synthesis pathway of *Cn. tall* was higher than that of *Cn. dwarf* (Fig. 4d). Moreover, the expression levels of the transcription factors MYB85, MYB58, and MYB46, which have been reported to regulate the metabolism and synthesis of lignin, were significantly different in various coconut tissues. Compared with *Cn. dwarf*, almost all of the three MYB transcription factors showed higher expression levels in *Cn. tall*, especially in the mesocarp. Among them, MYB46 has the highest expression level, indicating that it may play an important role in the discrepancy of lignin content between the two coconut varieties (Fig. 4e). Genomic and transcriptome analysis thus revealed that the number of genes related to lignin synthesis and regulation in *Cn. tall* and their gene expression levels were higher than those of *Cn. dwarf*.

To further study the biosynthesis and regulation of cellulose in coconut, we used the cellulose synthase CESA gene in *A. thaliana* for blast analysis. A total of 29 CESA genes were identified in coconut, including 15 in *Cn. tall* and 14 in *Cn. dwarf*, and phylogenetic tree analysis found that these CESA genes are divided into 6 groups (Fig. 4f). The number of CESA in *Cn. tall* in group 6 was more than that in *Cn. dwarf*, including the *AtCESA1* and *AtCESA10* genes that have been reported in *Arabidopsis* that were related to primary cell wall synthesis [31]. Compared with *Arabidopsis*, the CESA sequence of group 1 was quite different from that of *Arabidopsis*, which may be involved in the metabolism and synthesis of palm cellulose. We collected different tissues of *Cn. tall* and *Cn. dwarf*, including shoot tips, tender leaves, male flowers, copra, and mesocarp, and conducted transcriptome analysis. The results showed that the expression levels of all CEAS genes in different tissues of *Cn. tall* were higher than those of *Cn. dwarf* (Fig. 4g, Additional file 1: Figure S18). At the same time, the coconut leaves were dyed with phloroglucinol, revealing that the leaves of *Cn. tall* were more colored than *Cn. dwarf* (Fig. 4h). Thus via a range of genomic, transcriptomic, and cytological analysis, we proved that the cellulose content in *Cn. tall* is higher than that in *Cn. dwarf*. The above results also confirm from different levels that the fiber content in *Cn. tall* is more than that in *Cn. dwarf*.

### Multi-omics integration reveals the molecular mechanism of coconut plant height

**Fig. 5:**
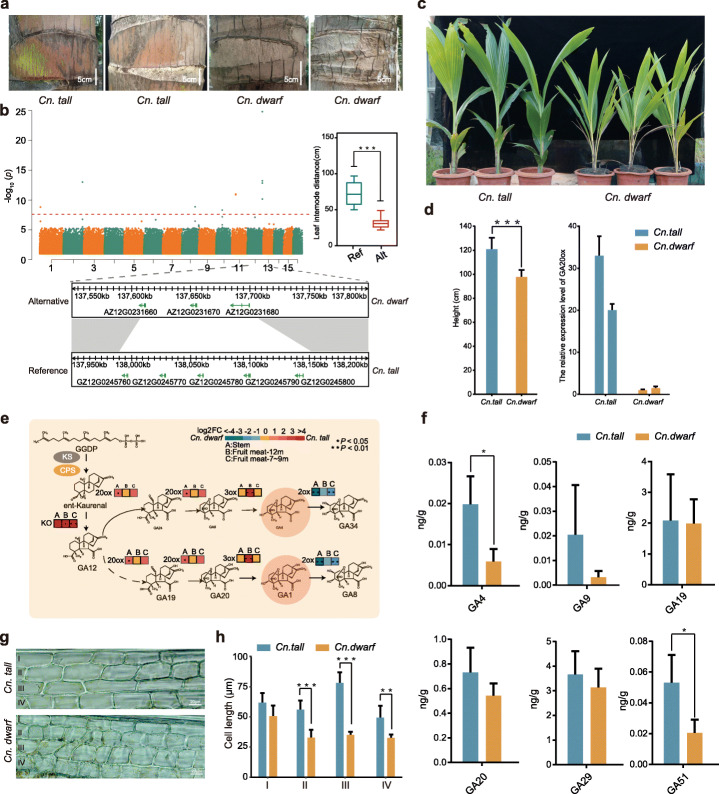
Multi-omics integrated analysis reveals the molecular basis of coconut plant height differences. **a** The leaf internode phenotype of *Cn. tall* and *Cn. dwarf*. **b** Genome-wide association analysis of the leaf internode phenotype of coconut. The upper panel shows the Manhattan plot of SNP-GWAS for leaf internode, and the lower panel shows copy number variation between *Cn. tall* and *Cn. dwarf* in chr12 chromosome, and the right panel is haplotype analysis of leaf spacing. *P* values were determined using two-tailed Student’s *t* test. **c** Images of *Cn. tall* and *Cn. dwarf* at seedling stage. **d** Comparison of height and GA20ox expression level by qRT-PCR between *Cn. tall* and *Cn. dwarf*. **e** Depiction of gibberellins metabolic pathway and gene expression bias between *Cn. tall* and *Cn. dwarf*. **f** Difference of gibberellin content in *Cn. tall* and *Cn. dwarf*. at seedling stage. **g** Optical microscope photographs of cells of *Cn. tall* and *Cn. dwarf*. **h** Differences in leaf cell length of *Cn. tall* and *Cn. dwarf* at seedling stage

Plant height is an important agronomic trait in coconut given that a squat phenotype is required for lodging resistance and mechanized harvesting, and indeed has been targeted as an important factor in establishing an ideal plant type with high yield. One of the most significant difference between the two subspecies of coconut is plant height. Eighty coconut varieties from different parts of the world were collected, including 13 *Cn. tall* and 67 *Cn. dwarf*, and phenotypic statistics were performed. The internode distance of “Hainan Tall” and “West African Tall” is significantly higher than that of the dwarf coconuts such as “Yellow Dwarf” and “Green Dwarf” (Fig. 5a), and there is a significant positive correlation between the internode distance and plant height of coconuts. To further explore the genetic mechanism of coconut plant height, we sequenced 80 coconut populations collected globally using next-generation sequencing technology, and obtained 60,150,070 SNPs, then 2,645,166 high-quality SNPs (MAF > 0.05, deletion rate < 10%, Additional file 22: Table S20) were selected to perform genome-wide association studies (GWAS). GWAS results identified a single genetic locus on Chr.12, which has a significant correlation with the internodes of the coconut population (*p* < 1.53e−25), indicating that the natural variation of plant height was mainly affected by this major locus (Fig. 5b, Additional file 1: Figure S19). The lead SNP was located in the promoter region of GA20ox (*GZ12G0245780*), so it was hypothesized that the SNP of GA20ox determines the phenotypic difference of internode. Furthermore, we found that the copy number of GA20ox in *Cn. tall* and *Cn. dwarf* is 5 and 3, respectively, and the SNP upstream of *GZ12G0245780* is closely related to the internode distance of coconut. Its genotype changed from type A to type G, and the internode of coconut increased significantly (*p* < 9.59e−26) (Fig. 5b). In addition, transcriptome analysis showed that the expression level of GA20ox gene on chromosome 12 of *Cn. tall* was higher than that of *Cn. dwarf*, and its fold change ranges from 1.3 to 12 (Additional file 1: Figure S20). Therefore, the copy number of GA20ox and the natural genetic variation of the SNP in the promoter caused the plant height difference of the coconut population.

In order to verify the influence of GA metabolism on coconut plant height, we collected young leaves of coconut seedlings for transcriptome, metabolome, and cytological analysis. The plant heights of *Cn. tall* and *Cn. dwarf* seedlings of the same age were significantly different, and RT-qPCR found that the GA20ox expression level of *Cn. tall* was significantly higher than that of *Cn. dwarf* (Fig. 5c, d). Transcriptome analysis revealed that there were significant differences in the overall expression of multiple key genes in the GA metabolic synthesis pathway in *Cn. tall*. Compared with *Cn. dwarf*, the overall expression of GA synthesis genes such as *ent*-kaurene oxidase (KO), GA20ox, and GA3ox in tall coconuts is significantly higher, especially the KO gene expression in *Cn. tall* which is 16 times higher than that of *Cn. dwarf*. At the same time, we found that the expression of GA2ox in the GA degradation pathway in *Cn. tall* was significantly lower than that in *Cn. dwarf*, and the total expression of this gene in *Cn. dwarf* was 2–16 times that of *Cn. tall* (Fig. 5e). In addition, we conducted metabolomic analysis of young coconut leaves and found that the content of multiple intermediates in the GA synthesis pathway in *Cn. tall* is higher than that in *Cn. dwarf*, especially the content of GA4, which is biologically active (Fig. 5f). The length of the leaf cells of *Cn. tall* is significantly longer than that of *Cn. dwarf* (Fig. 5g, h). Based on the above results, we found that coconut plant height is mainly affected by the structural variation of GA20ox, and evidences such as transcriptome, metabolome, and cytology have further confirmed the difference mechanism of coconut plant height.

## Discussion

Here we performed the sequencing and assembly of two high-quality coconut reference genomes through a combination of nanopore sequencing, Hi-C and Illumina technologies, covering of its two main ecotypes: *Cn. tall* and *Cn. dwarf*. Considering that two coconut genomes were sequenced in different Nanopore platforms with different mean sequencing read length, the assembled N50 contigs were 14.3 and 2.9 Mb for *Cn. dwarf* and *Cn. tall*, respectively, which are at least 201- and 40-fold higher than the previous reported coconut genomes [15, 32]. Although there are enormous differences of phenotypic traits between *Cn. tall* and *Cn. dwarf*, including height, flowering time, fruit color, fruit size, fruit yield, oil content, germination time and ratio, cellulose content, pollination pattern, and fiber percentage, the genomes from these two coconut types revealed high similarity and collinearity (Fig. 3). Nonetheless, thousands of PAVs and large SVs were detected by comparing the genomes of *Cn. tall* and *Cn. dwarf*. It is very possible that SVs included the candidate genes that result in the variation of targeted traits [33–35]. Two large deletions in *Cn. dwarf* compared to *Cn. tall* may well be associated with the divergence of germination time and rates, and cellulose contents between *Cn. tall* and *Cn. dwarf*.

To study the formation and evolution of the coconut genome, collinearity analysis with the oil-free camphor genome found that the coconut genome has undergone two genome-wide replication events during its evolution. The *Ks* distribution of palms such as coconut, oil palm, and date palm indicated that WGD occurred before their lineage differentiations [21, 36] (Fig. 1c). Moreover, phylogenetic analysis showed that a common WGD *τ* event (approximately 129–145 Mya) was detected in monocot species. The *C. nucifera* genome had another WGD event (approximately 47–53 Mya) before it differentiated from *E. guineensis* and *P. dactylifera*, and it had the highest similarity with *E. guineensis*, with a divergence time of 17–19 Mya, *and* a divergence time 2–8 Mya between *Cn. tall* and *Cn. dwarf*. Here, we reconstructed the 10 proto-chromosomes of ancient monocot and the evolutionary trajectories leading to the formation of the extant chromosomes of coconut and several other monocot plants. Our inference is different from previous reports of 5 or 7 ancestral monocot chromosomes [26, 37]. The present inference used a well-assembled coconut genome sequence decoded by third-generation sequencing technology, and considered not only the gene collinearity and classification of multiple syntenic blocks, but also the telomere-centric genome repatterning model [38]. This model which emphasizes that telomeres had to be removed to result in fusion of different chromosomes, was implemented to reconstruct the proto-chromosomes and their evolutionary trajectories in different groups of plants [39, 40].

Wild coconuts spread globally especially to China through ocean currents, and the content of coconut fiber is an important factor affecting the spread of coconuts across oceans. Compared with nuts of the cultivated *Cn. dwarf*, nuts of the *Cn. tall* can drift on the sea due to their large size and high fiber content of the coconuts facilitating the inter-continental spread of coconuts. The fiber of coconut is mainly composed of cellulose and lignin, and the high degree of fibrosis of the coconut stem makes the analysis of gene transcription level and metabolite content very difficult. Multi-omics comparative analysis of tender tissues of different types of coconuts revealed that the number and expression of genes for cellulose and lignin synthesis and regulation in *Cn. tall* are higher than those in *Cn. dwarf*. Indeed, the high fiber content of tall coconuts likely created conditions for the transoceanic spread of coconuts, while the breeding behavior of humans pursuing coconut meat and coconut milk has negatively selected the fiber content, resulting in the low fiber content of dwarf coconuts.

Plant height is the most important trait to consider in the coconut breeding process, since it is a crucial factor determining both yield and harvesting efficiency. After transoceanic propagation, the human-driven breeding process pursued coconut yield and more convenient picking methods, thus artificially selecting for reduced plant height and eventually forming dwarf coconut. In previous studies, the plant hormone “GA” had been demonstrated to be able to promote the cell and internode elongation, which subsequently make plant higher [41–44]. Based on the high-quality coconut genome and resequencing data in this study, we used the leaf internode length phenotype of adult coconut trees to perform GWAS and found that GA20ox gene on Chr12, the ortholog of the gene that drove the Green Revolution, affects the plant height traits of coconut. Previous results reported that GA20ox showed high expression level in leaf blade, leaf sheath, stem, and unopened flower [45]. Therefore, we analyzed the differences in GA20ox expression and GA content in different tissues such as tender leaves, flowers, stem tips, and flesh of the two coconuts based on multi-omics, and revealed the effect of GA metabolism and synthesis on the differences in coconut plant height. The copy number and natural variation of GA20ox can thus be used as molecular markers for the selection and identification of coconut varieties, thereby speeding up the process and efficiency of coconut breeding. The differentiation of the coconut plant height means that the selection of plant height over millions of years has been aimed at the same target gene, not only in the natural selection of ancient plant as illustrated for coconut, but also in the artificial selection of heavily cultivated crops such as rice and maize. Taken together, the two high-quality coconut reference genomes reported in this study provide insights into the evolutionary history of monocot chromosomes, the molecular basis of key agronomic important traits, genomics of fiber formation linked with transoceanic spread of coconuts, and the molecular mechanism underlying height of coconut ecotypes. The finding that nature pre-empted the strategy we much later adopted to drive the Green Revolution is fascinating. It opens up massive opportunities given that in contrast to, for example, cereals for which yield increases are stagnating this could represent a general approach for increasing the yield of less widely cultivated properties. Given that in coconut, large populations can be made a propagated in vitro bypassing, the need for multiple rounds of selfing such as required for cereals. This approach will thus not only potentially lead to greater yield enhances but also dramatically shorten the time taken to achieve this. The results presented here thus represent a comprehensive resource for future functional genomics and molecular breeding studies of this important tropical resource.

## Conclusions

In this study, we provided two high-quality reference-grade genomes of *Cn. tall* and *Cn. dwarf*, with contig N50s of 2.9 Mb and 14.3 Mb, respectively. The high-quality coconut genome and its key nodes in the evolution of monocots allowed us to reconstruct the ancestral karyotypes of the 10 chromosomes of monocots, and thereby reconstructed the evolutionary trajectories of the 16 coconut chromosomes. The genome sequence alignment between *Cn. tall* and *Cn. dwarf* reveals that a large segment deletion (0.12–0.18 Mb) on the chromosome 4 and 12 may be associated with the divergence of plant height and cellulose content. Intriguingly, fiber synthesis genes in *Cn. tall*, main genes related to lignin and cellulose synthesis, have a higher copy number and expression level than dwarf coconuts. Integrated multi-omics analysis revealed that the difference in coconut plant height is affected by gibberellin metabolism and that the GA20ox copy number and a single-nucleotide change in the promoter together led to the difference in plant height between *Cn. tall* and *Cn. dwarf*. Traditional breeding techniques are still difficult for coconuts because of its long lifecycle. We can analyze the genetic basis of key traits such as plant height and fiber content through multi-omics methods, and develop relevant molecular markers based on the genetic variation of their traits. Moreover, modern molecular marker breeding technology will speed up the process of coconut nutrient quality improvement and precise breeding of new varieties. In total, this research provides a large amount of genomic information, which will promote the research of coconut functional genomics and molecular marker-assisted breeding.

## Methods

### Plant material

We independently evaluated spear leaf, female flower, male flower, and shoot apical meristem tissue harvested from *Cn. tall* and *Cn. dwarf* for transcriptome and metabolome analysis. The *Cn. tall* and *Cn. dwarf* seedlings were grown at seedling nursery of Coconut Research Institute (Wenchang City, Hainan Province, China). The spear leaf and shoot apical meristem were collected from seedlings half a year after germination, while female and male flowers were collected from 15-year-old grown *Cn. tall* and *Cn. dwarf* tree from the National Coconut Germplasm Nursery of Coconut Research Institute. Meanwhile, in order to identify the candidate genes associated with the height divergence between *Cn. tall* and *Cn. dwarf*, eighty 15-year-old coconut individuals, 13 tall coconuts, and 67 dwarf coconuts were selected from National Coconut Germplasm Nursery of Coconut Research Institute and their spear leaves were collected for DNA extraction and subsequently resequencing.

### Transcriptome and metabolome analysis

Total RNA was extracted separately from the different tissues of coconut individual plants using the Invitrogen Trizol kit. Purified mRNA isolated, in three biological replicates, from different tissues were separately fragmented with divalent cations under increased temperature. These short fragments were used as templates to synthesize the first-strand cDNA using hexamer primers and superscript^TM^III (Invitrogen^TM^, Carlsbad, CA, USA). Second-strand cDNA was then synthesized in a solution containing buffer, dNTP, RNaseH, and DNA polymerase I and subsequently purified using a QiaQuick PCR extraction kit (Qiagen). EB buffer was used to resolve these short fragments for end reparation and poly (A) addition. The sequence adaptors were linked to two ends of short cDNA sequences, and suitably sized cDNA fragments were selected out for PCR amplification based on the agarose gel electrophoresis results. Finally, the library established was sequenced using an Illumina Hiseq^TM^ 2000. The paired-end library was developed according to the Paired-End sample Preparation kit protocol (Illumina, USA). According to published procedures, the assembly of transcriptome short reads was completed based on the reference genome using transcriptome assembly software [46]. FPKM (fragments per kilobase per million) was used to calculate gene expression level by using RSEM software [47].

For metabolic analysis, the fresh coconut samples were collected from individual plants, and then immediately frozen in liquid nitrogen, and stored at − 80 °C until needed. A plant sample with a fresh weight of 50 mg was ground into powder under liquid nitrogen and extracted with 500 μl of H_2_O/ACN. Before extraction, the internal standard lidocaine was added to the plant samples. The supernatant after centrifugation was collected, and the above steps to extract the residue again are repeated. Then, 10 μl of triethylamine (TEA) and 10 μl of 3-bromopropyltrimethylammonium bromide (BPTAB) were added to the resulting solution. The reaction solution was vortexed, incubated at 90 °C for 1 h, then evaporated to dryness in a nitrogen stream, then dissolved in 100 μl H_2_O/ACN, and filtered through a 0.22-μm filter for further LC-MS analysis. The sample extracts were analyzed using an LC-ESI-MS/MS system (UHPLC, ExionLC™ AD; MS, Applied Biosystems 6500 Triple Quadrupole, https://sciex.com.cn/).

### DNA sequencing library construction and sequencing

Genomic DNA was extracted using a QIAGEN® Genomic DNA extraction kit (Cat#13323, QIAGEN) according to the standard operating procedure provided by the manufacturer. The extracted DNA was detected by a NanoDrop™ One UV-Vis spectrophotometer (Thermo Fisher Scientific, USA) for DNA purity (OD260/280 ranging from 1.8 to 2.0 and OD 260/230 is between 2.0 and 2.2), then Qubit® 3.0 Fluorometer (Invitrogen, USA) was used to quantify DNA accurately. After that, long DNA fragments were selected and collected using the BluePippin system (Sage Science, USA). Recycled DNA fragments were subjected to end repair and adaptor ligation by SQK-LSK109 kit. The final libraries were sequenced by the Nanopore PromethION platform (Oxford Nanopore Technologies, UK).

### Estimation of the genome size and heterozygosity

The genome size was estimated by *k*-mer frequency analysis. The distribution of *k*-mer depends on the characteristic of the genome and follows Poisson’s distribution. Before assembly, the 19-mer distribution of 126 Gb Illumina short reads was generated using Jellyfish (v2.2.6) [48] and was uploaded to the GenomeScope website (http://qb.cshl.edu/genomescope/). As a result, we obtained an estimated haploid *Cn. dwarf* genome size of 2.38 Gb with a 0.16% heterozygous rate.

### Hi-C-based genome assembly

Both *Cn. tall* and *Cn. dwarf* genomes were assembled de novo using SMARTdenovo (https://github.com/ruanjue/smartdenovo) [-k 19 -J 3000] based on Nanopore long reads corrected with NextDenovo (https://github.com/Nextomics/NextDenovo). Raw contigs were polished using Nanopore long reads and Illumina short reads with NextPolish (v1.2.2) [49]. Then polished contigs were anchored into chromosomes by using HiC-Pro (v2.11.1) [50] and LACHESIS [51]. Finally, the assembled genomes were manual correction with Juicebox (v1.11.08).

To assess the quality of assembled genomes, raw Illumina paired-end reads and RNA-seq reads from multiple tissues were mapping to the genomes using BWA (v0.7.17-r1188) [52] and HISAT2 (v2.1.0) [53], respectively. In addition, Benchmarking Universal Single-Copy Orthologs (BUSCO) was also used to assess the genome completeness base on Embryophyta Plant database (odb10) [54].

### Genome annotation

LTR_FINDER (v1.07) [55], MITE-Hunter [56], and RepeatModeler were used to build a de novo transposable element (TE) library. Then, the Repbase database [57] and the de novo library were combined to obtain a consensus library. RepeatMasker was applied to identify transposable elements and mask the genome sequences from the constructed consensus library. And the masked genomes were used for gene annotation.

Gene annotation of *C. nucifera* genome was conducted by combining de novo prediction, homology information, and RNA-seq data. First, Augustus (v3.2.3) [58] and GenScan (v1.0) [59] were used on the repeat masked genome with trained parameters. For homology-based gene prediction, the non-redundant proteins from four sequenced species, *E. guineensis*, *P. dactylifera*, *M. acuminate*, and *A. comosus*, from Phytozome (http://www.phytozome.net), were used to infer the annotation of protein-coding genes in *C. nucifera* genome by GeMoMa (v1.6.1) [60]. Furthermore, RNA-seq data were mapped to the genome using TopHat2 (v2.1.1) [61], and StringTie (v1.3.0) [62] was used to assemble transcripts to gene models. Finally, all predictions were combined with EVidenceModeler (EVM; v1.1.1) [63] to get a non-redundant gene set, and PASA (v2.3.0) [64] was used to correct the predicted result and annotate alternatively spliced isoforms to finalize the gene set. Functional annotation of protein-coding genes was evaluated by BLASTP (v2.7.1+) [65] with an *E*-value cutoff of 1 × 10^−5^ using two integrated protein sequence databases—SwissProt and TrEMBL [66]. Protein domains were annotated by searching InterPro and Pfam databases, using InterProScan (v5.25) [67] and Hmmer (v3.2.1) [68], respectively. Gene ontology terms for each gene were obtained from the corresponding InterPro or Pfam entry. The pathways, in which the gene might be involved, were assigned by BLAST against the Kyoto Encyclopedia of Genes and Genomes (KEGG) [69] database.

### Genome polyploidization analysis

Eight representative plants were adopted to perform comparative genomics analysis, to understand the occurrence and (non-)sharing of polyploidization event(s), and to infer evolutionary trajectories to form extant chromosomes. These plants included seven monocots: *C. nucifera* in this study, *E. guineensis*, *S. polyrhiza*, *A. officinalis*, *M. acuminate*, *A. comosus*, *O. sativa*, and one eudicot, *V. vinifera* (Additional file 23: Table S21). Colinear blocks, genomic regions containing colinear genes, within each genome and between them were inferred using ColinearScan (v.1.0) [70] according to the combined information of gene similarity and gene order. Putative homologous genes were predicted by using BLASTP, with *E*-values < 1e−5, and used as input of the software. As previous reports, a relative loose *E*-value threshold would not jeopardize the inference of colinear genes, the authenticity of which was further ensured by their shared orders on different chromosomes. Maximal gap between genes involved in collinearity along a chromosome was set to be 50 intervening or non-colinear genes. To help date evolutionary events and identify colinear genes produced by different events, polyploidization or speciation, synonymous substitutions per synonymous site (*Ks*) between colinear genes were estimated using the Nei–Gojobori approach as implemented in the PAML package (v4.9) [71].

Plants were found to often evolve with different rates, especially after polyploidization. Here, we had similar findings and adopted a genomics approach to perform evolutionary rate correction by using *Ks* inferred between colinear genes. The genomics approach had been used previously and could be briefed as follows: firstly, the median *Ks* was calculated for each colinear block, and the probability density distribution curve of *Ks* was estimated using MATLAB with the kernel smoothing density function (ksdensity; bandwidth was typically set to 0.025). Multipeak fitting of the curve was performed using the Gaussian approximation function (cftool) in MATLAB. Secondly, based on *Ks* distribution of *O. sativa*, *Ceratophyllum demersum*, and *V. vinifera* in a previous study [72], the *Ks* correction coefficient of species were calculated and got the corrected *Ks* rate. Then the corrected *Ks* was used to date evolutionary events with the same rate.

### Inferring ancestral cell karyotypes and reconstruct chromosome evolutionary trajectories

By referring to gene collinearity between compared species, we found that *S. polyrrhiza* (Sp) and *A. comosus* (Ac) provided great opportunity to infer ancestral karyotypes of basal monocots, due mainly to their well-preserved gene collinearity. For example, gene collinearity showed prominent homology between the middle part of Sp chromosome 1 (Sp1), the middle part of Sp6, the former part of Sp7, and the former part of Sp12, which were surely produced by two rounds of polyploidizations (*α* and *β*) (Additional file 1: Figure S13c); these listed regions in Sp chromosomes further corresponded to the middle and latter part of Ac chromosome 1 (Ac1), the middle part of Ac5, nearly whole chromosome Ac17 and chromosome Ac21, and the majority of Ac20. The paralogs between these Ac chromosomes were produced by sigma (*σ*) whole-genome triplication (WGT) (Fig. 2b, Additional file 1: Figure S15), which are shared across the Poales plants. Actually, the above chromosome homology supported that they were derived from ancestral chromosomes M4 and M5 (Fig. 2a, Additional file 1: Figure S15), which were inferred to have been produced by duplicating the proto-chromosome A1 since the node A before the split with *S. polyrrhiza*. By characterizing gene collinearity between *S. polyrrhiza* and *C. nucifera*, *A. officinalis*, and *V. vinifera*, respectively, the evolutionary trajectory of proto-chromosome A1 obtained further support. The evolutionary trajectory of proto-chromosome A3 was deduced by analyzing gene collinearity of *S. polyrrhiza* (Sp) and *A. comosus* (Ac), from which we found that the latter part of Sp2, Sp3, and the latter part of Sp7 (produced by *α* and *β*) corresponded to one group of chromosomes (Ac4, Ac13, and Ac14) and the other one group (Ac12, Ac18, and Ac23). Besides, several chromosomes were directly inherited from the proto-chromosomes. For example, the extant chromosomes Sp15, Sp13, Sp16, Sp20, Ac24, and Ac25 were derived directly from proto-chromosomes A6, A7, A8, A9, A10, and A4, respectively. While the evolutionary trajectories of A2 and A5 were more complex, each of its corresponding duplicated chromosomes was reorganized with the other chromosomes derived from proto-chromosomes (Additional file 1: Figure S15).

### SNP and InDel calling and structural variation analysis between *Cn. tall* and *Cn. dwarf*

MUMmer4 (v4.0.0) was used to compare the genomes of *Cn. tall* and *Cn. dwarf*, and one-to-one alignment blocks were identified by delta-filter with parameter -1. Then show-snps was used to identify SNPs and InDels (< 100 bp) with parameter -ClrTH. The snpEff software was used to annotate the effects of SNPs and InDels. The potential PAVs between *Cn. tall* and *Cn. dwarf* were identified using show-diff in MUMmer4 Toolkit. To further verify the results, the candidate PAVs were mapped to the genomes using BLASTN and the PAVs which coverage > 80% were filtered out to obtain the final PAV regions. The gene having > 80% overlap with PAV regions was considered to be a PAV-related gene. Furthermore, SyRI [73] was used to identify large structure variations greater than 1000 bp in length.

### Gene family analysis and phylogenetic reconstruction

Protein sequences of the longest transcripts in 17 species, including *Cn. tall*, *Cn. dwarf* (this study), *E. guineensis*, *P. dactylifera*, *C. simplicifolius*, *D. jenkinsiana*, *S. bicolor*, *P. persica*, *S. tuberosum*, *G. max*, *A. thaliana*, *T. cacao*, *V. vinifera*, *M. acuminata*, *C. papaya*, *P. trichocarpa*, and *N. colorate*, from Phytozome (http://www.phytozome.net), were used to identify gene families by OrthoFinder (v2.3.11) [74]. A total of 249 single-copy orthologous genes were obtained from the step of gene family analysis. Then, amino acid sequences encoded by single-copy genes from these 17 species were aligned using MUSCLE (v3.8.1551) [75], and RaxML (v8.2.12) [76] was used to construct a phylogenetic tree based on the result of multiple sequence alignments. The MCMCTree program in PAML (v4.9) [71] was used to estimate the divergence time of all the 17 species, with previously published calibration time (*A. thaliana* and *C. papaya* was 54–90 Mya, *A. thaliana* and *P. trichocarpa* was 98–117 Mya) [77]. CAFÉ (v4.2) [78] was used to calculate the expansion and contraction of gene family numbers based on the phylogenetic tree and gene family statistics.

### SNP genotyping and genome-wide association analyses

The 80 coconut accessions used in this study were characterized by whole-genome resequencing. Fastp software was used for data quality detection and filtering with default parameters. The clean reads were aligned to *Cn. tall* genome with BWA software [52]. PCR duplicates were removed with Picard java program. The Genome Analysis Toolkit (GATK v4.0) was used to variant calling with the HaplotypeCaller module, the command is “-R reference.fa -I input.bam -O GCVF -ERC GVCF -ploidy 2 -stand_call_conf 30.0.” GVCF files were merged with the “GenotypeGVCFs” command. SNPs and InDels were filtered out if their mapping quality < 20 or their sequencing depth < 50 in the whole population. Population structure was modeled as a random effect in EMMA using Admixture software, the kinship (K) matrix was calculated by emmax-kin, and the parameter is emmax-kin -v -h -s -d 10. Efficient Mixed-Model Association eXpedited (EMMAX) software was used to conduct all associations [79]. The effective number of independent SNPs was calculated using Genetic type 1 Error Calculator (GEC) software [80]. The calculated genome-wide significance thresholds, based on a nominal level of 0.05, were *P* = 2.56 × 10^− 8^ for the whole population. LD decay was calculated based on the *R*^2^ value by Plink (v1.9). Furthermore, to reduce the redundancy of GWAS signals, the lead SNP within 1 Mb window was extracted as a single signal.

### Web links and URLs

Food and Agricultural Organization of the United Nations (FAO) statistics, http://faostat.fao.org/; RepeatModeler, http://www.repeatmasker.org/RepeatModeler/; RepeatMasker, http://www.repeatmasker.org/RepeatMasker/; MUMmer4, https://github.com/mummer4/mummer.

## Supplementary Information

**Additional file 1: Figs. S1-S20**. **Fig. S1.** Images and genome size of coconut. **Fig. S2.** Heatmaps for Hi-C assembly in *Cn. tall*. **Fig. S3.** Heatmaps for Hi-C assembly in *Cn. dwarf*. **Fig. S4.** *Cn. tall* and *Cn. dwarf* Hi-C. **Fig. S5.** Distribution and divergence of TEs and associated PAVs. **Fig. S6.** Genome duplication, Ks distribution, and evolutionary dating. **Fig. S7.** Homologous dotplots within each Arecaceae genome. **Fig. S8.** Homologous gene dotploting between coconut and the other Arecaceae genomes. **Fig. S9.** Ks of collinear genes within each Arecaceae genome. **Fig. S10.** GO enrichment analysis of expansive and contracted gene families. **Fig. S11.** Analysis of coconut and other species gene family. **Fig. S12.** Homologous genes plot of coconut. **Fig. S13.** Homologous gene plots. **Fig. S14.** The inferred evolutionary trajectories to form extant *Spirodela polyrhiza* chromosomes. **Fig. S15.** The inferred evolutionary trajectories to form extant *Ananas comosus* chromosomes. **Fig. S16**. Genome variation between *Cn. tall* and *Cn. dwarf*. **Fig. S17.** Enrichment analysis of specific genes. **Fig. S18.** The expression pattern of CESA gene in different tissues of coconut. **Fig. S19.** Quantile-quantile plot for height phenotype. **Fig. S20.** Statistics on change fold of GA20ox expression on Chr. 12 with *Cn.tall* compare *Cn.dwarf*.

**Additional file 2: Table S1.** Raw sequencing data.

**Additional file 3: Table S2.** Mapping summary of Hi-C data.

**Additional file 4: Table 3.** Anchored chromosome length of *Cn. tall* and *Cn. dwarf* genome.

**Additional file 5: Table S4.** Evaluation assembled genome based on Illumina PE reads.

**Additional file 6: Table S5.** Evaluation of *Cn. tall* and *Cn. dwarf* genomes based on different tissues RNA-seq.

**Additional file 7: Table S6.** Evaluation of assembly completeness with respect to genespace using BUSCO.

**Additional file 8: Table S7.** Statistics of transposable element.

**Additional file 9: Table S8.** Gene function annotation statistics.

**Additional file 10: Table S9.** Statistics of annotated non-coding RNAs.

**Additional file 11: Table S10.** Kernel function analysis of Ks distribution related to duplication events within each genome and between genomes (before evolutionary rate correction).

**Additional file 12: Table S11.** Kernel function analysis of Ks distribution related to duplication events within each genome and between genomes (after evolutionary rate correction).

**Additional file 13: Table S12.** Kernel function analysis of Ks distribution related to duplication events within each genome (before evolutionary rate correction).

**Additional file 14: Table S13.** Orthology of protein-coding genes among 15 species and *Cocos nucifera*.

**Additional file 15: Table S14.** The information of homologous genes between *C. nucifera* and other four species.

**Additional file 16: Note S1.** More information about inferring ancient monocot karyotypes and evolutionary trajectories to form extant chromosomes.

**Additional file 17: Table S15.** The syntenic blocks detected between *Cn. tall* and *Cn. dwarf* by MUMmer.

**Additional file 18: Table S16.** The syntenic blocks detected between *Cn. tall* and *Cn. dwarf* by MCScanX.

**Additional file 19: Table S17.** The summary of SNPs and InDels.

**Additional file 20: Table S18.** The summary of PAVs.

**Additional file 21: Table S19.** The summary of large chromosome structural variation between *Cn. tall* and *Cn. dwarf*.

**Additional file 22: Table S20.** The phenotypic data of internode distance among coconut population.

**Additional file 23: Table S21.** Information of reported genome data.

**Additional file 24.** Review history.

## Data Availability

The whole-genome sequence data have been deposited in the Genome Warehouse in National Genomics Data Center, Beijing Institute of Genomics, Chinese Academy of Sciences/China National Center for Bioinformation, under the accession number GWHBEBT00000000 [81] and GWHBEBU00000000 [82], which are publicly accessible at https://ngdc.cncb.ac.cn/gwh. And other raw sequence data have been deposited in the Genome Sequence Archive in National Genomics Data Center, with the accession number CRA004778 [83] and CRA005120 [84], which are publicly accessible at https://ngdc.cncb.ac.cn/gsa.
