# Supplementary material for: High-quality reference genome sequences of two coconut cultivars provide insights into evolution of monocot chromosomes and differentiation of fiber content and plant height
Source: Genome Biol. 2021 Nov 4;22:304. doi: 10.1186/s13059-021-02522-9 (PMC8567702; doi:10.1186/s13059-021-02522-9)
Supplement: Supplementary file 1 — Additional file 1: Figs. S1-S20. Fig. S1. Images and genome size of coconut. Fig. S2. Heatmaps for Hi-C assembly in Cn. tall. Fig. S3. Heatmaps for Hi-C assembly in Cn. dwarf. Fig. S4. Cn. tall and Cn. dwarf Hi-C. Fig. S5. Distribution and divergence of TEs and associated PAVs. Fig. S6. Genome duplication, Ks distribution, and evolutionary dating. Fig. S7. Homologous dotplots within each Arecaceae genome. Fig. S8. Homologous gene dotploting between coconut and the other Arecaceae genomes. Fig. S9. Ks of collinear genes within each Arecaceae genome. Fig. S10. GO enrichment analysis of expansive and contracted gene families. Fig. S11. Analysis of coconut and other species gene family. Fig. S12. Homologous genes plot of coconut. Fig. S13. Homologous gene plots. Fig. S14. The inferred evolutionary trajectories to form extant Spirodela polyrhiza chromosomes. Fig. S15. The inferred evolutionary trajectories to form extant Ananas comosus chromosomes. Fig. S16. Genome variation between Cn. tall and Cn. dwarf. Fig. S17. Enrichment analysis of specific genes. Fig. S18. The expression pattern of CESA gene in different tissues of coconut. Fig. S19. Quantile-quantile plot for height phenotype. Fig. S20. Statistics on change fold of GA20ox expression on Chr. 12 with Cn.tall compare Cn.dwarf. [file 13059_2021_2522_MOESM1_ESM.docx]

**Supplementary Figures**

High-quality reference genome sequences of two coconut cultivars provide insights into evolution of monocot chromosomes and differentiation of fiber content and plant height

Shouchuang Wang^1,2,3,a^, Yong Xiao^1,4,a^, Zhi-Wei Zhou^5,6,a^, Jiaqing Yuan^7,a^, Hao Guo^2,a^, Zhuang Yang^2,3^, Jun Yang^2^, Pengchuan Sun^8^, Lisong Sun^2,3^, Yuan Deng^2,3^, Wen-Zhao Xie^5^, Jia-Ming Song^6^, Muhammad Tahir ul Qamar^6^, Wei Xia^2^, Rui Liu^1^, Shufang Gong^1^, Yong Wang^1^, Fuyou Wang^1^, Xianqing Liu^2^, Alisdair R. Fernie^9^, Xiyin Wang^8,b^, Haikuo Fan^1,b^, Ling-Ling Chen^6,b^, Jie Luo^2,3,b^

^1^Hainan Key Laboratory of Tropical Oil Crops Biology, Coconut Research Institute, Chinese Academy of Tropical Agricultural Sciences, Wenchang, China

^2^College of Tropical Crops, Hainan University, Haikou, 570228, China

^3^Sanya Nanfan Research Institute of Hainan University, Hainan Yazhou Bay Seed Laboratory, Sanya, 572025, China

^4^Sanya Research Institute of Chinese Academy of Tropical Agricultural Sciences, Sanya, China

^5^National Key Laboratory of Crop Genetic Improvement, Huazhong Agricultural University, Wuhan, 430070, China

^6^State Key Laboratory for Conservation and Utilization of Subtropical Agro-bioresources, College of Life Science and Technology, Guangxi University, Nanning 530004, China

^7^College of Life Sciences, Shanxi Normal University, Xi’an, 710119, China

^8^Center for Genomics and Computational Biology, North China University of Science and Technology, Tangshan, China

^9^Max Planck Institute of Molecular Plant Physiology, Potsdam-Golm 14476, Germany

^a^These authors contributed equally to this work

^b^To whom correspondence should be addressed. E-mail: jie.luo@hainanu.edu.cn or llchen@gxu.edu.cn or venheco@163.com or wangxiyin@vip.sina.com


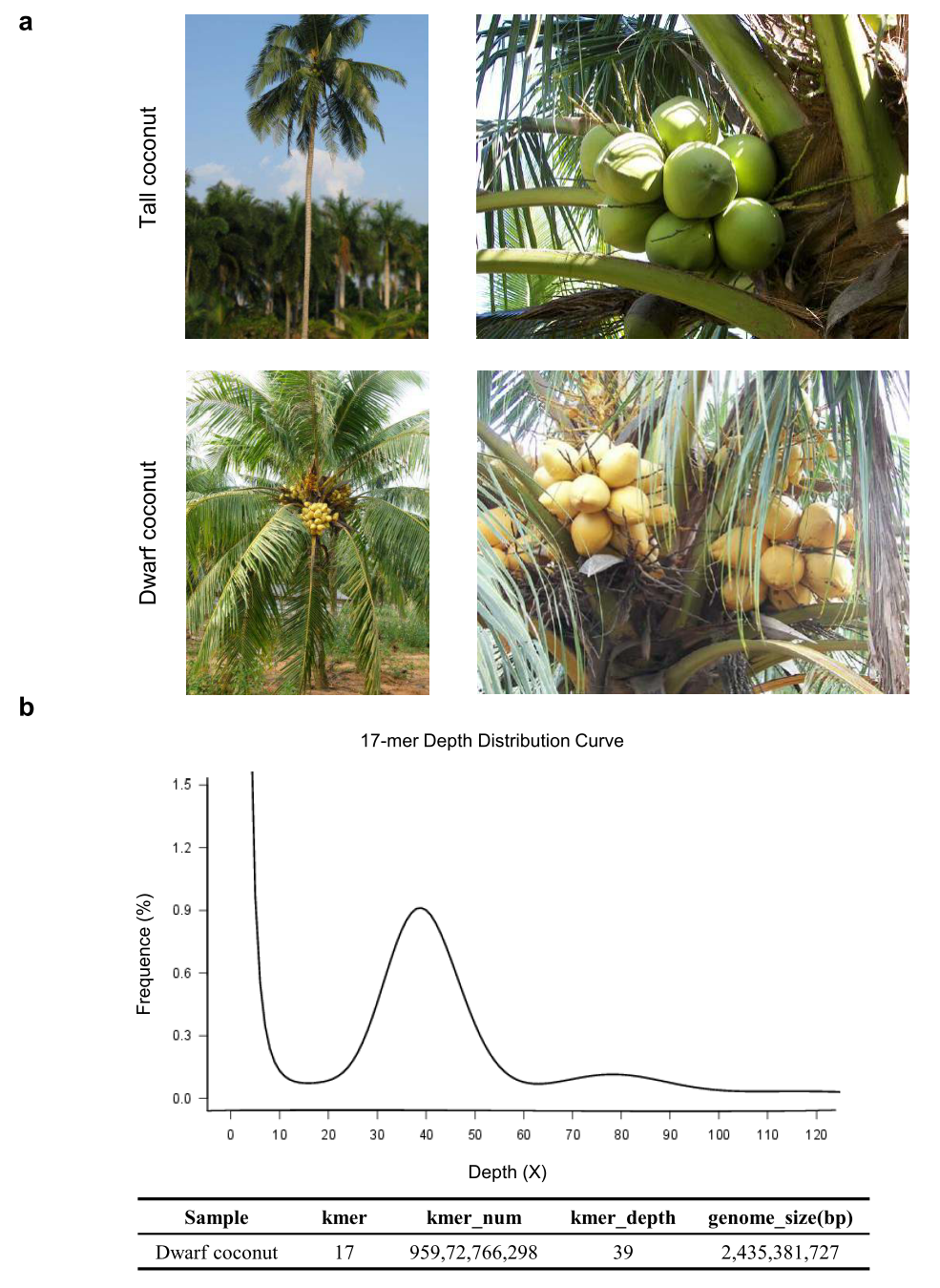


**Fig S1.** **Images and genome size of coconut.**

**a**, Images of whole coconut plant and fruit. **b**, Genome size estimation from K-mer analysis for dwarf coconut. The 17-mer frequency distribution of dwarf coconut is showed about. The genome size is estimated using the formula: G = N × (L - K + 1) / K_depth, where N represents the total of number of reads, L represents the read length, K represents the k-mer value used in the analysis, and K depth refers to the main peak in the k-mer distribution curve.


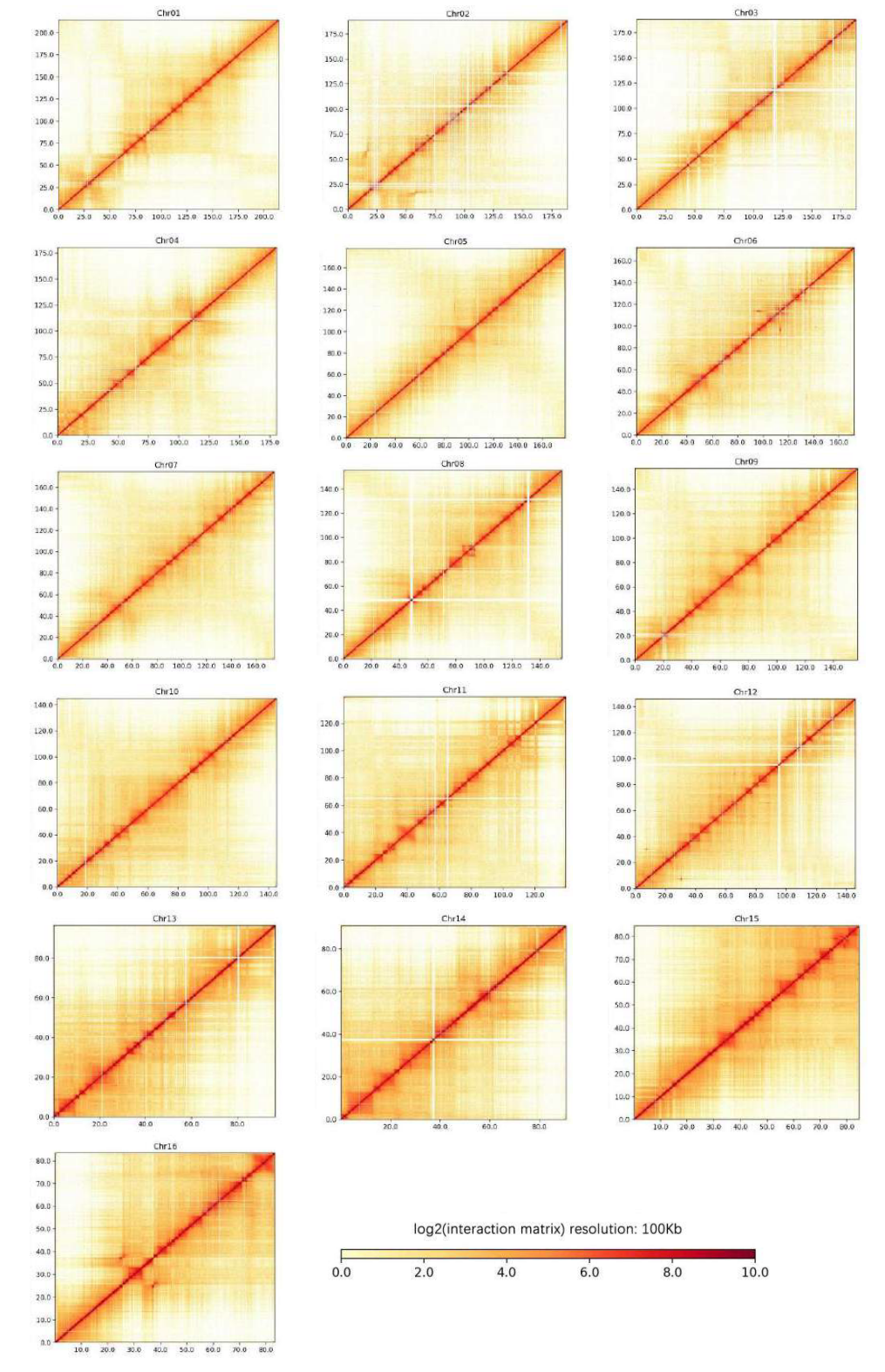


**Fig S2.** Heatmaps for Hi-C assembly in *Cn. tall.*

Each heatmap is shown at a resolution 100 kb. The dots from light yellow to dark red show low to high probability of interactions.


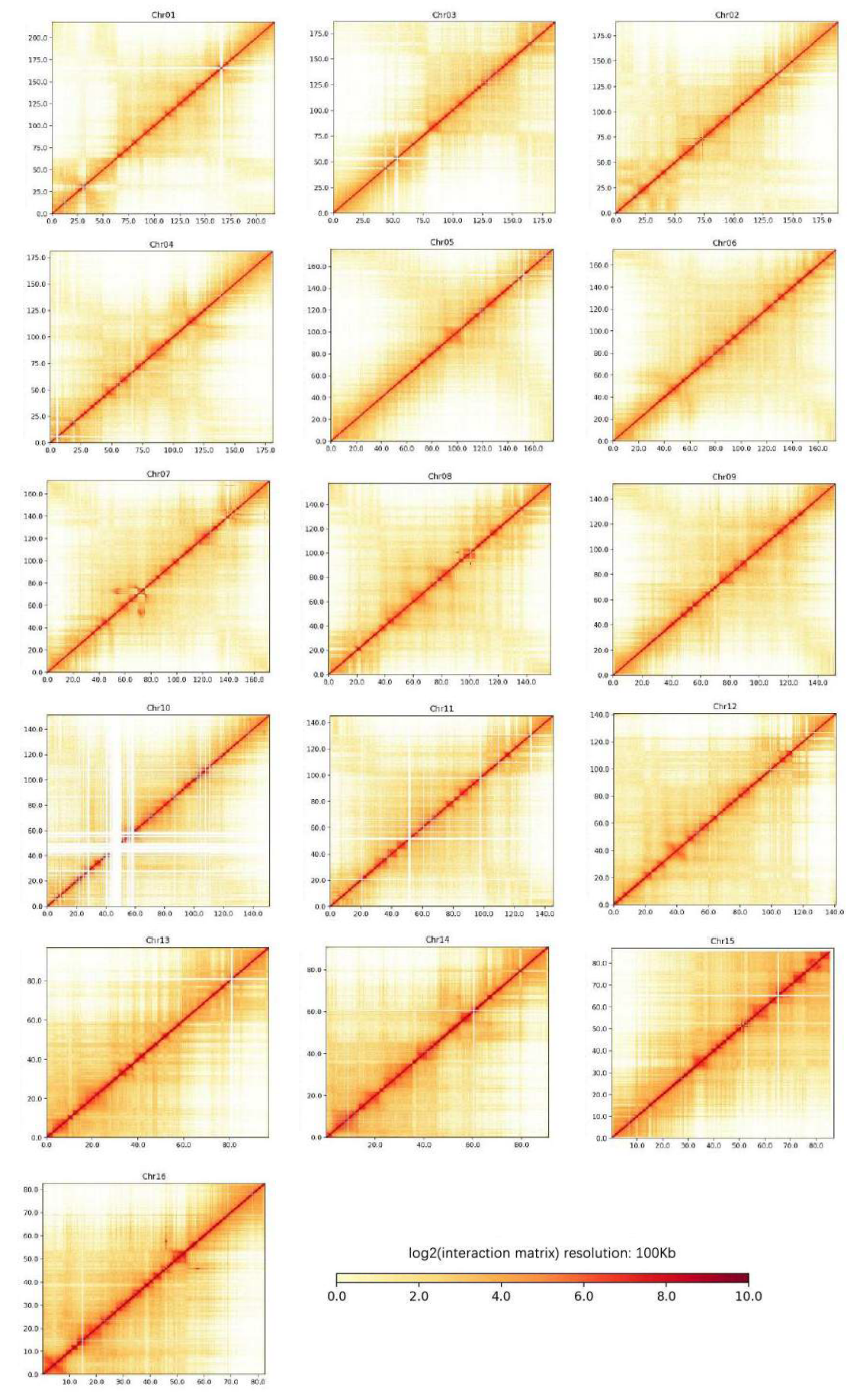


**Fig S3.** Heatmaps for Hi-C assembly in *Cn. dwarf*.

Each heatmap is shown at a resolution 100 kb. The dots from light yellow to dark red show low to high probability of interactions.


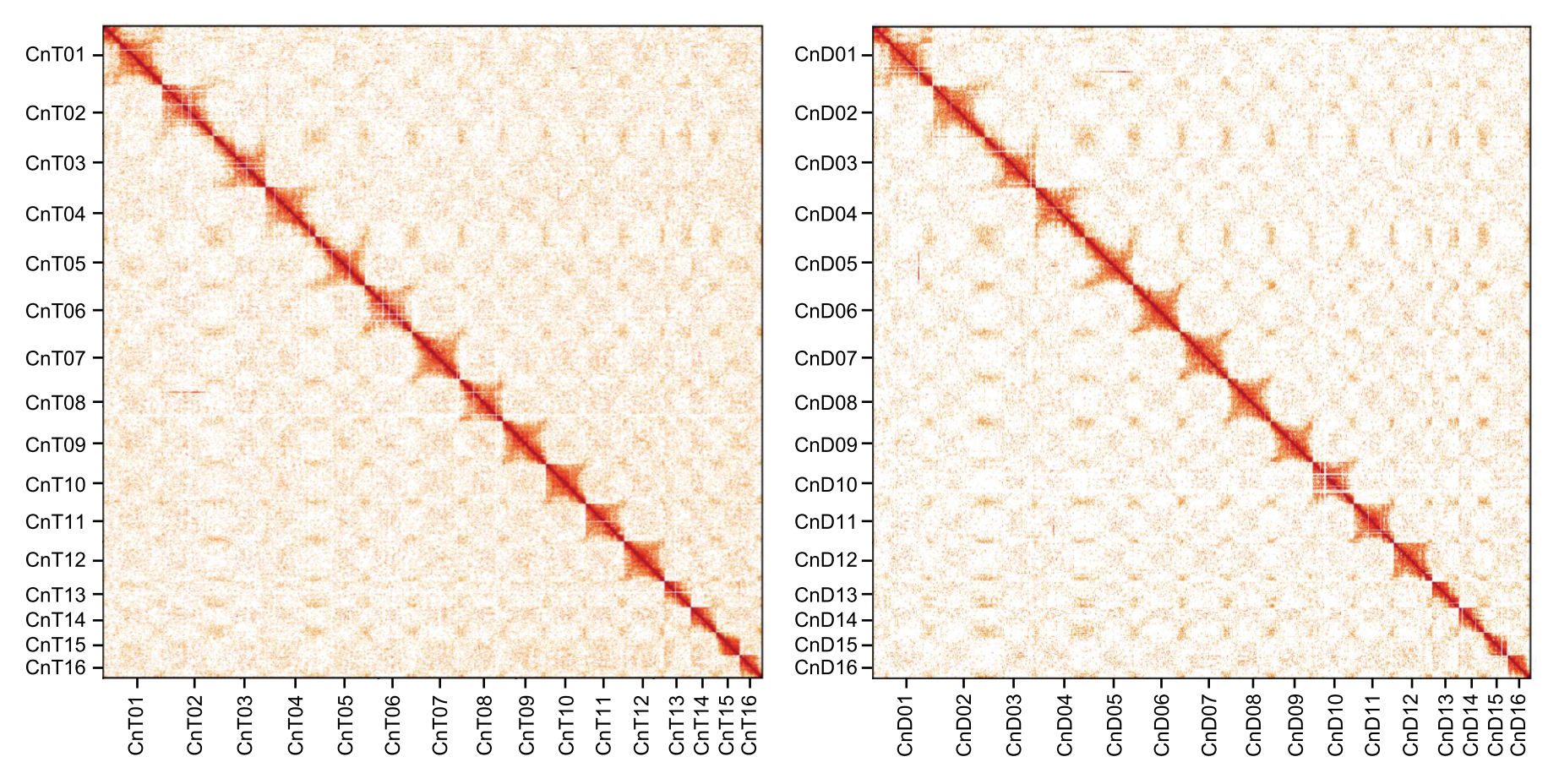


**Fig S4.** ***Cn. tall* and *Cn. dwarf* Hi-C.**

The color intensity shows the frequency of contact between two 500 kb loci.


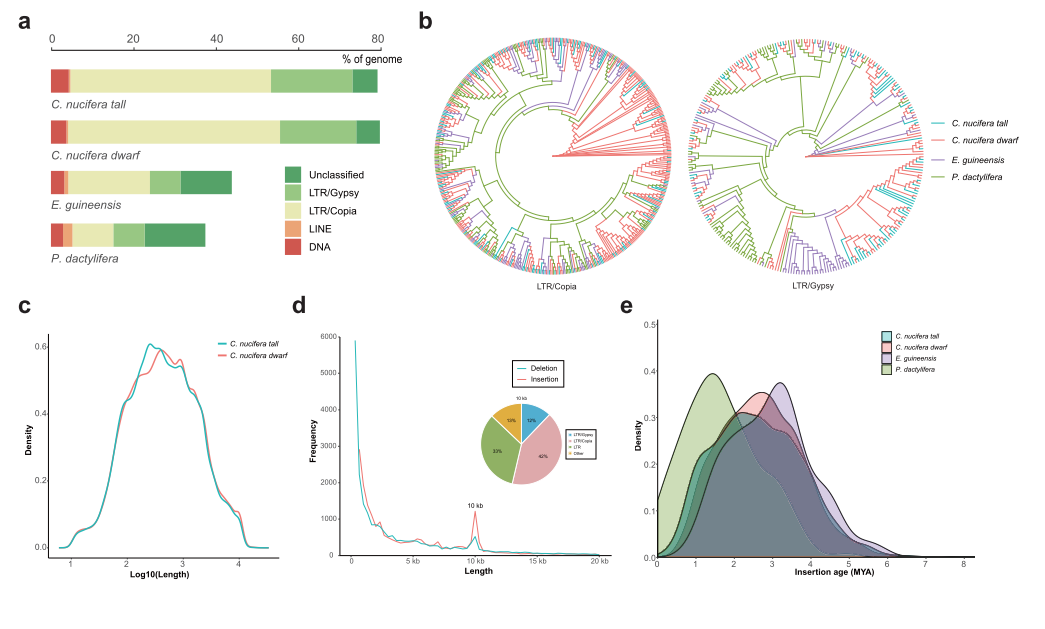
 **Fig S5. Distribution and divergence of TEs and associated PAVs.**

**a**, Percentage of classified TEs in *Cn. tall*, *Cn. dwarf*, *E. guineensis*, and *P. dactylifera*. **b**, Phylogenetic relationship of LTR/Copia and LTR/Gypsy in *Cn. tall*, *Cn. dwarf*, *E. guineensis* and *P. dactylifera*. **c**, The length distribution of TEs in *Cn. tall* and *Cn. dwarf* genomes. **d**, The pie chart shows the composition of TEs in about 10 kb peak. **e**, Distribution of LTR insertion time in *Cn. tall*, *Cn. dwarf*, *E. guineensis* and *P. dactylifera*.


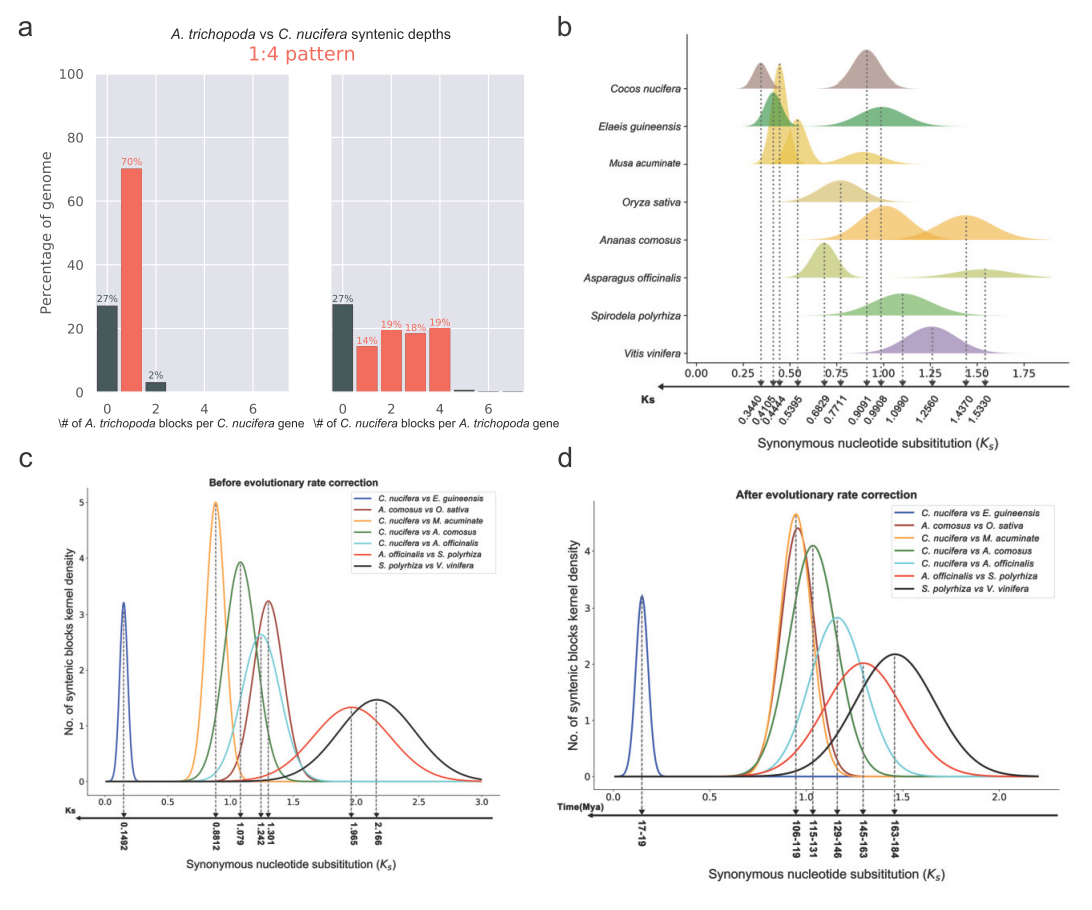


**Fig S6. Genome duplication, Ks distribution, and evolutionary dating.**

**a**, The distribution of syntenic depths between *A. trichopoda* and *C. nucifera*. **b**, Density distributions of the Ks values for paralogous genes before evolutionary rate correction. **c**, Density distributions of the Ks values for orthologous genes before evolutionary rate correction. **d**, Density distributions of the Ks values for orthologous genes after evolutionary rate correction.


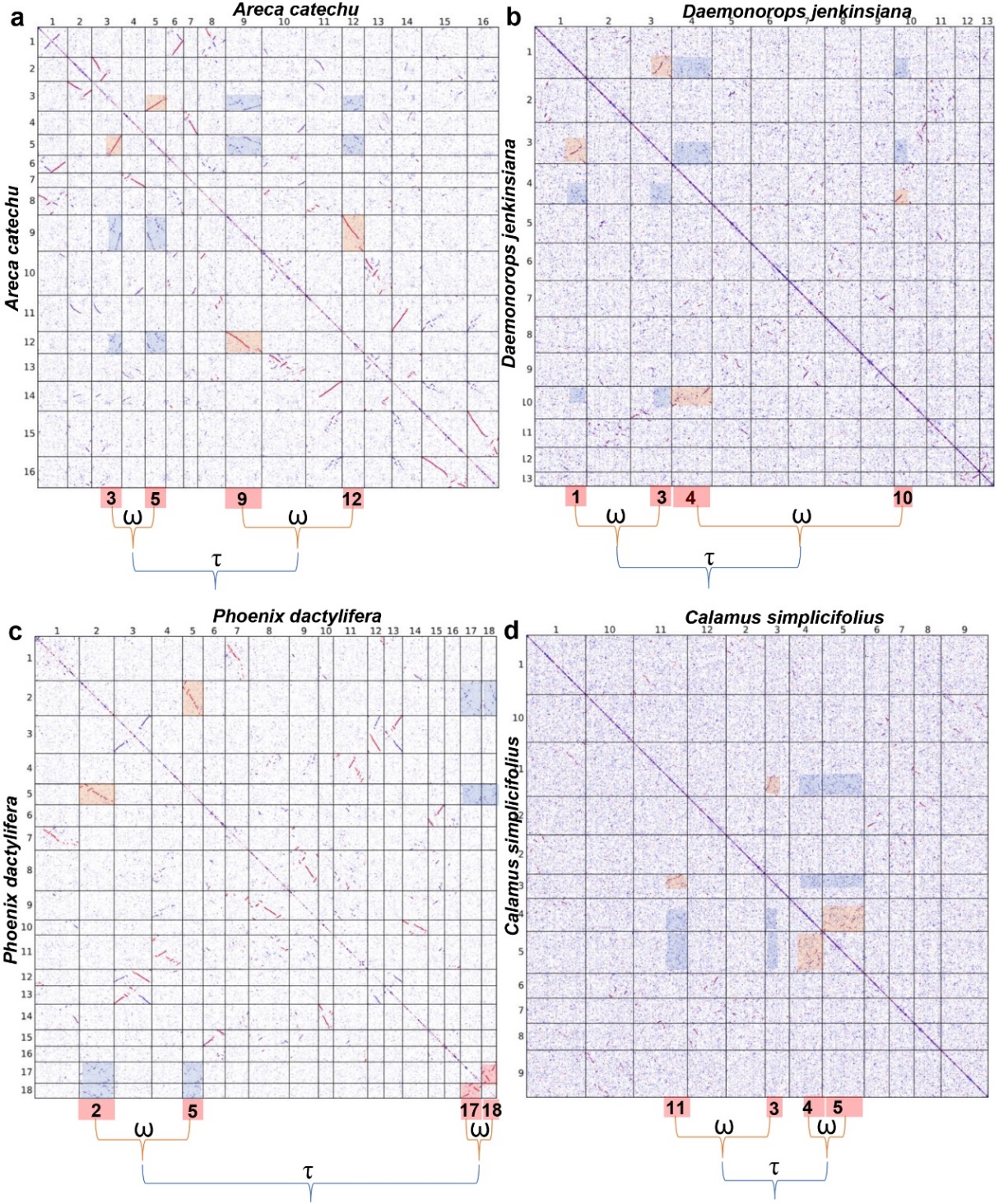


**Fig S7. Homologous dotplots within each Arecaceae genome.**

The red dots show their best-matched genes, blue dots secondarily matched ones, and the gray ones being produced by more ancient or dispersal duplicated genes. The regions surrounded by red frames were likely produced by **ω** WGD; those surrounded by blue frames were likely produced by **τ** WGD. **a-d**. homologous gene dotplotting within each of *Areca catechu*, *Phoenix dactylifera*, *Daemonorops jenkinsiana,* and *Calamus simplicifolius*.


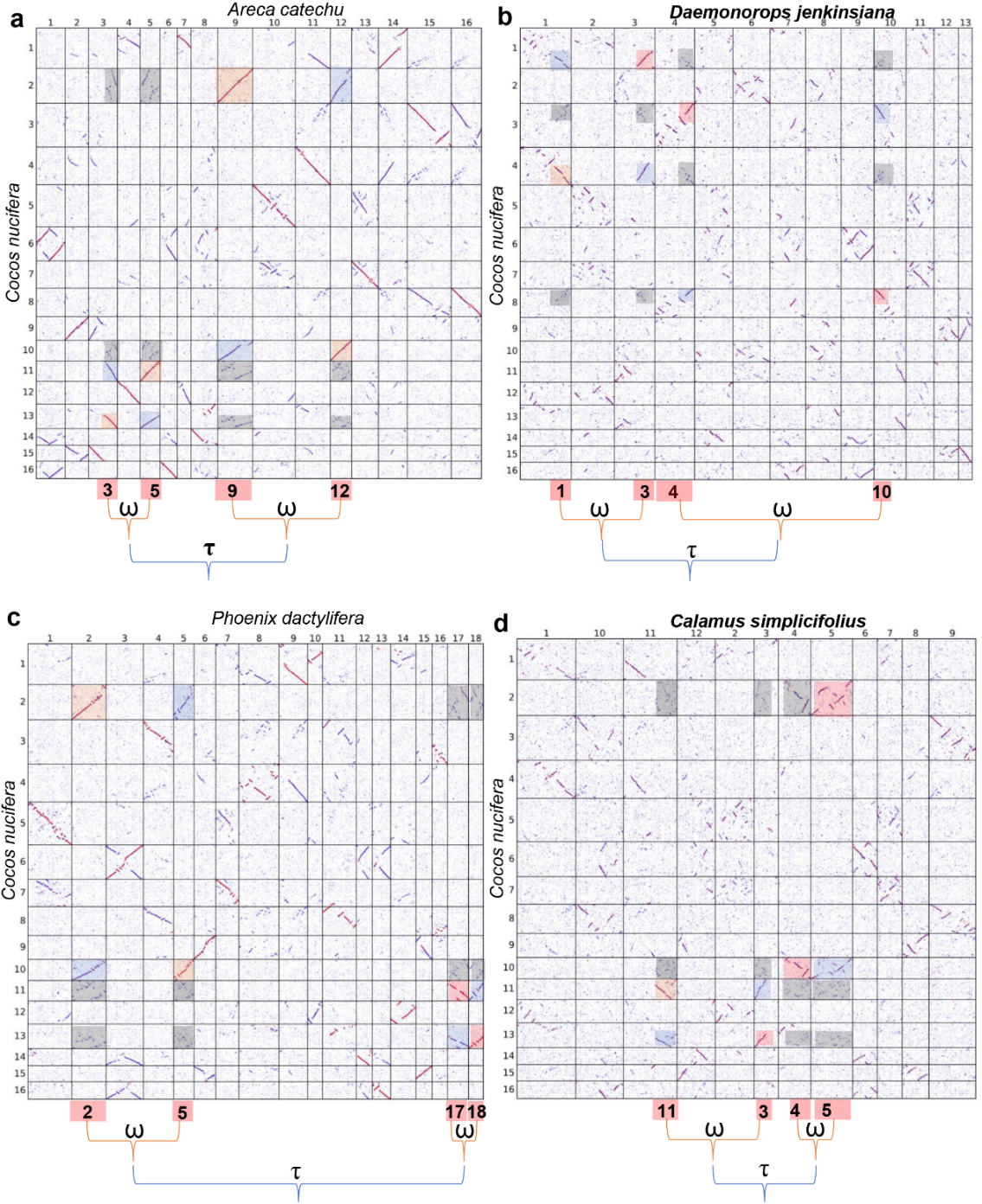


**Fig S8. Homologous gene dotploting between coconut and the other Arecaceae genomes.**

The red dots show their best-matched genes, blue dots secondarily matched ones, and the gray ones being produced by more ancient or dispersal homologs. The regions surrounded by red frames show likely orthologous correspondence. The correspondence between chromosomes or chromosome regions between two plants were likely produced by WGD **ω or τ**, indicated below each dotplot. **a-d**. Homolgous gene dotplots of *Areca catechu*, *Phoenix dactylifera*, *Daemonorops jenkinsiana,* and *Calamus simplicifolius* with coconut (*Cocos nucifera*).


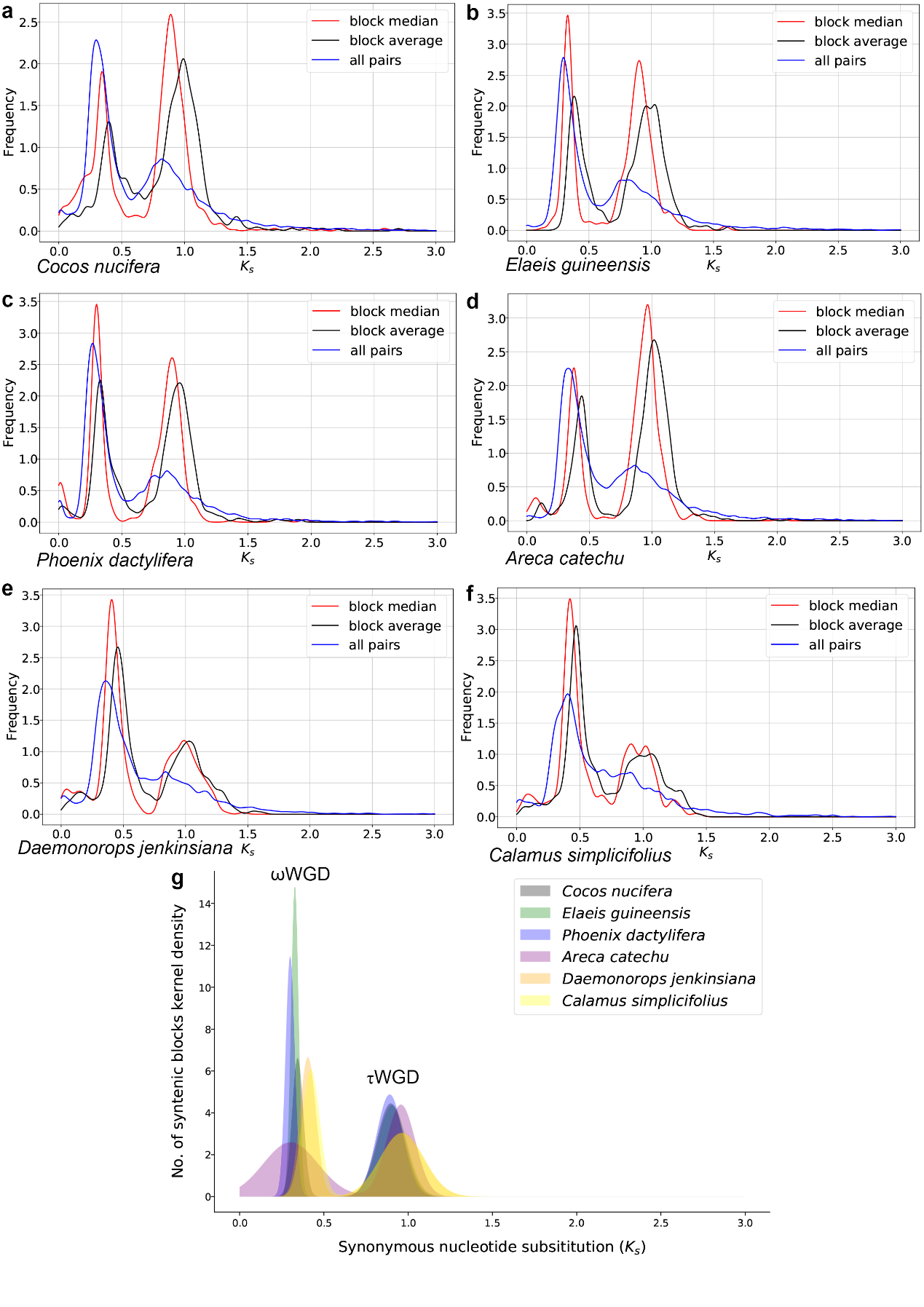


**Fig S9. Ks of collinear genes within each Arecaceae genome.**

The distribution of Ks between all collinear genes, the average and medians in each duplicated blocks containing the collinear genes were shown. **a-f**. *Cocos nucifera****,*** *Elaeis guineensis*, *Areca catechu, Phoenix dactylifera, Daemonorops jenkinsiana and Calamus simplicifolius*, **g.** A merged Ks distributions with clusters of two peaks each indicating two WGDs ω and τ, respectively. The Ks distribution were based on median values from duplicated blocks, and subjected to statistical operation with kernel function.


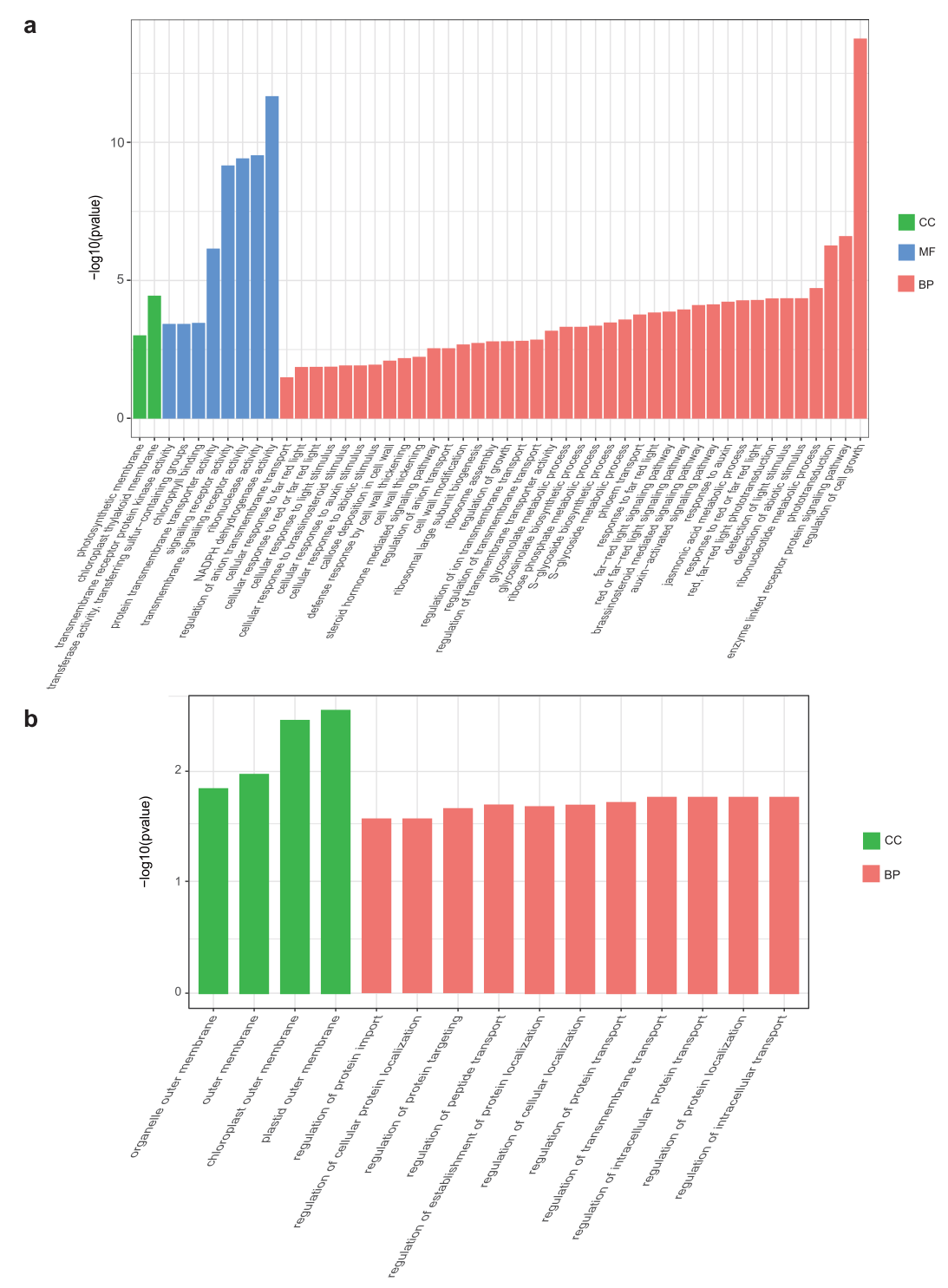


**Fig S10. GO enrichment analysis of expansive and contracted gene families.**

**a**, Enrichment analysis of the expansive genes in *Cn. tall*. All 1,019 expansive gene families were clustered to 50 GO categorizes. **b**, Enrichment analysis of the contracted genes in *Cn. tall*. All 1,574 contracted gene families were clustered to 15 GO categorizes. CC indicates cellular component, MF indicates molecular function, BP indicates biological process.


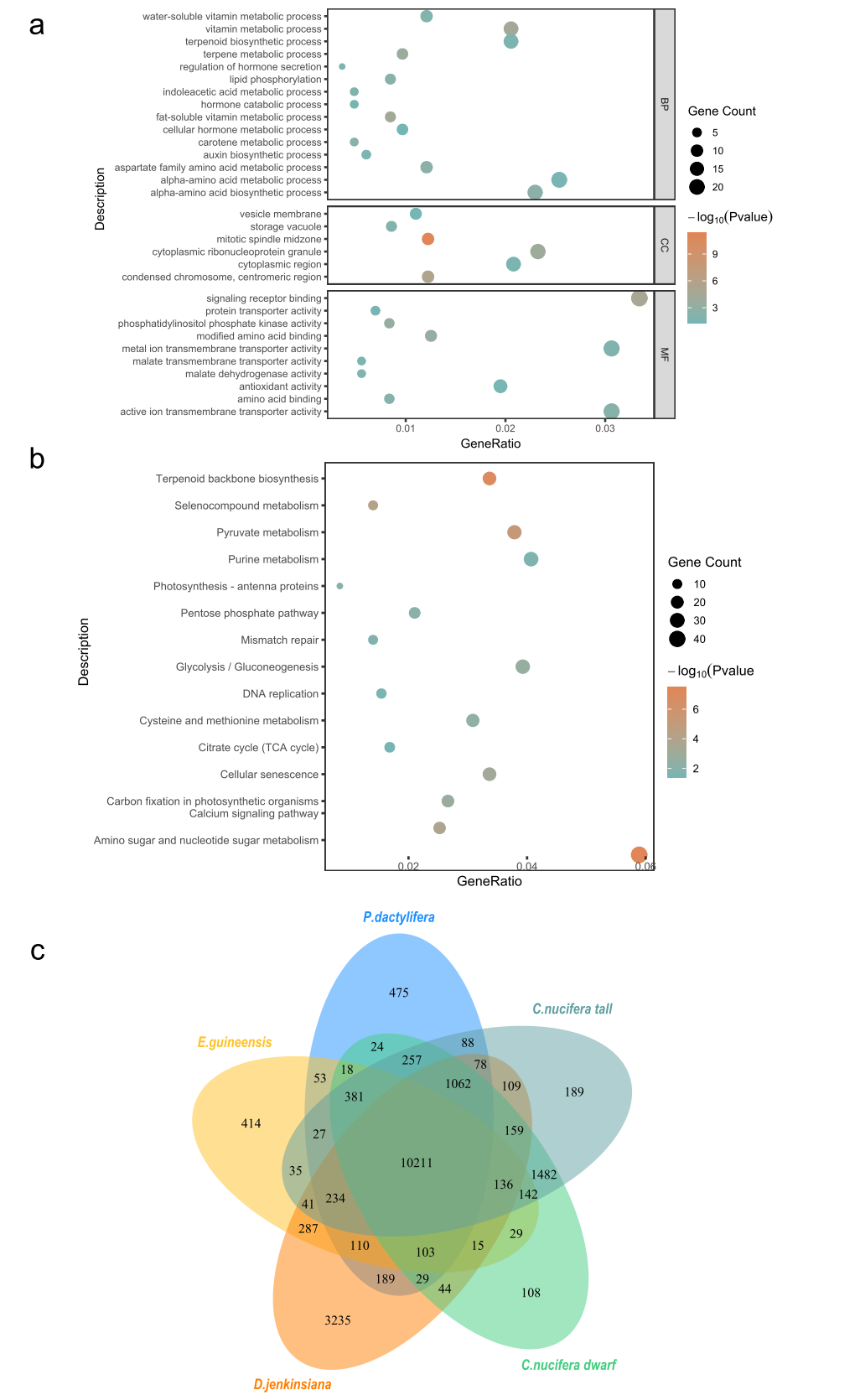


**Fig S11. Analysis of coconut and other species gene family.**

**a** and **b**, GO and KEGG enrichment analysis of *C. nucifera* specific gene families. **c**, Clusters of orthologous and paralogous gene families in *Cn. tall*, *Cn. dwarf*, and three palm species.


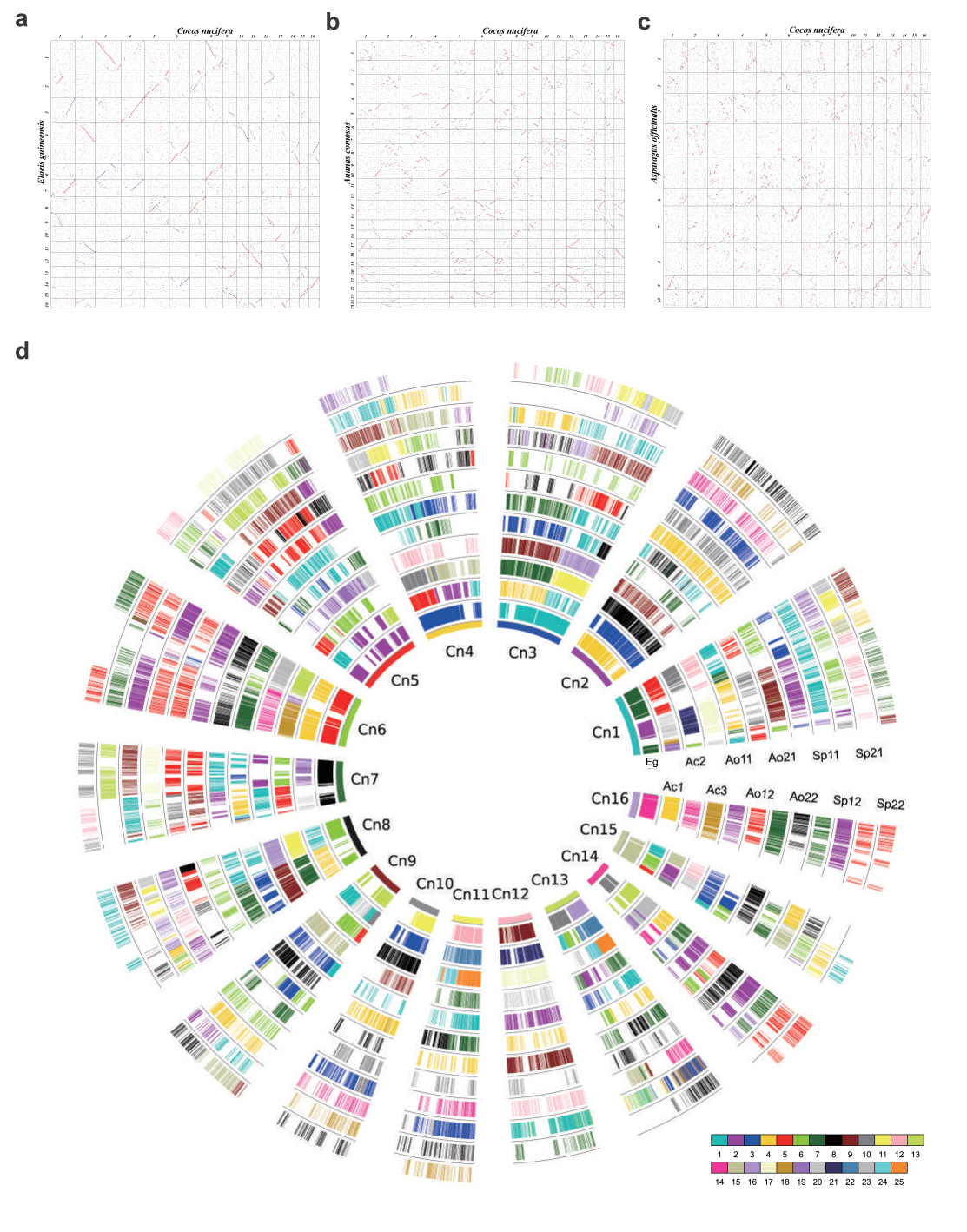


**Fig S12. Homologous genes plot of coconut.**

The homologous dotplot of *C. nucifera* and *E. guineensis* (**a**), *A. comosus* (**b**), *A. officinalis* (**c**). **d**. Global alignment of homologous regions in *C. nucifera* (Cn), *E. guineensis* (Eg), *A. comosus* (Ac), *A. officinalis* (Ao) and *S. polyrhiza* (Sp).


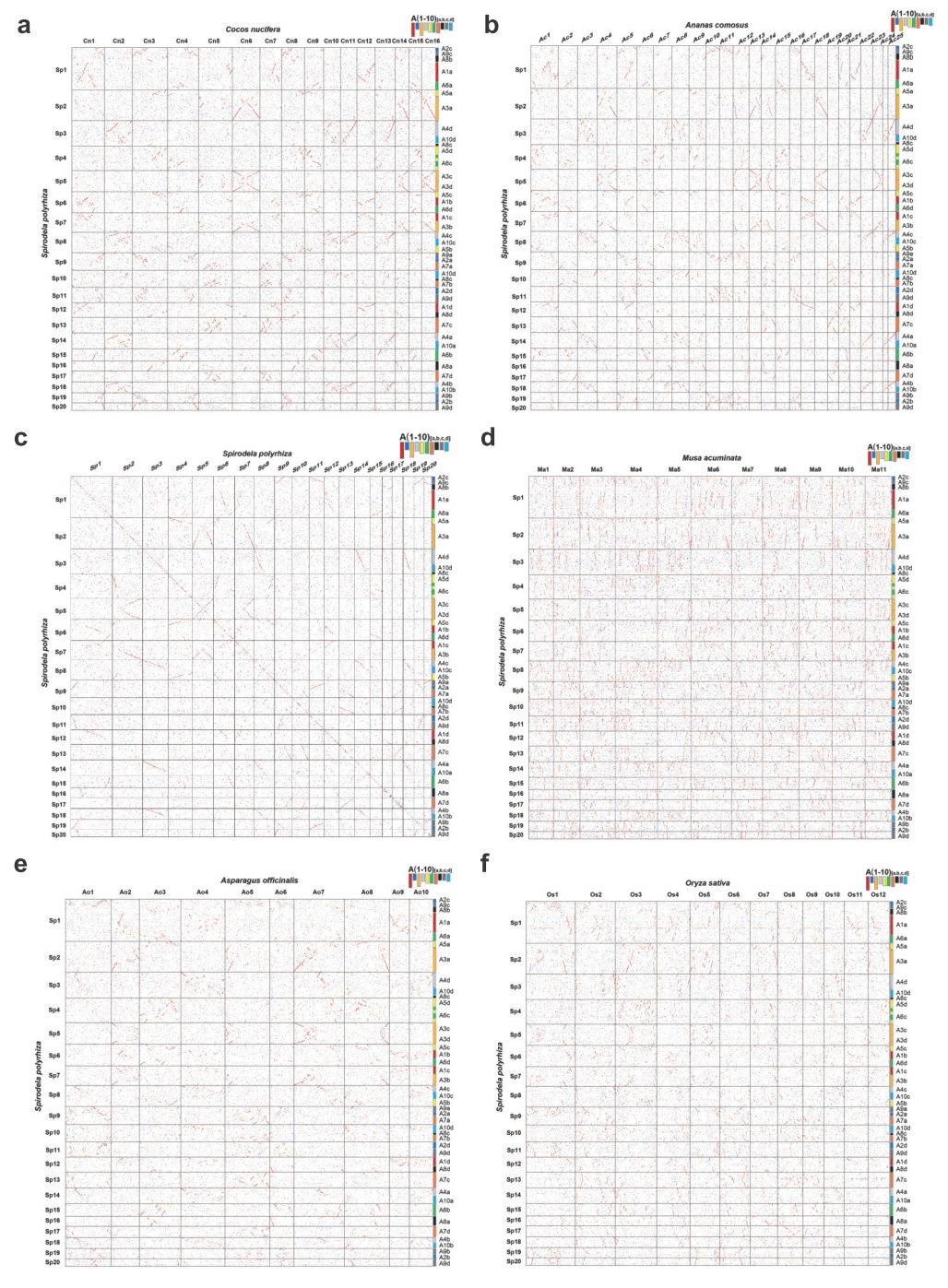


**Fig S13. Homologous gene plots.**

The homologous dotplots of *S. polyrhiza* and *C. nucifera* (**a**), *A. comosus* (**b**), *S. polyrhiza* (**c**), *M. acuminata* (**d**), *A. officinalis* (**e**), *O. sativa* (**f**). The correspondence of the extant chromosomes to 10 proto-chromosomes at node A, each showing in a specific color, were denoted besides each dotplot figure.


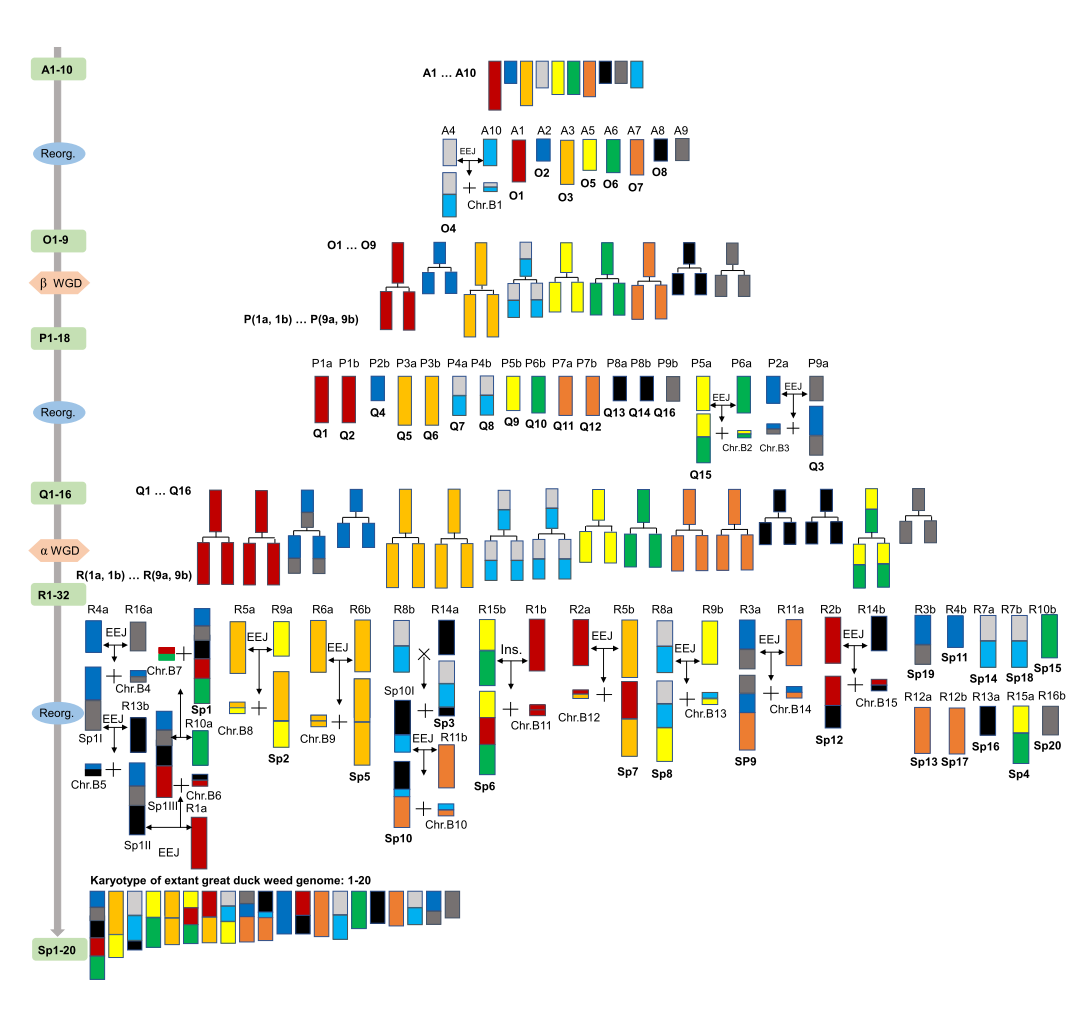


**Fig S14. The inferred evolutionary trajectories to form extant *Spirodela polyrhiza* chromosomes.**

The sign of “EEJ” represents “end end join”; the sign of “Reorg.” represents reorganization; the sign “Ins.” represents insertion. the sign of “Chr. B” represents mini-chromosome (B chromosome) produced by the “EEJ” process.


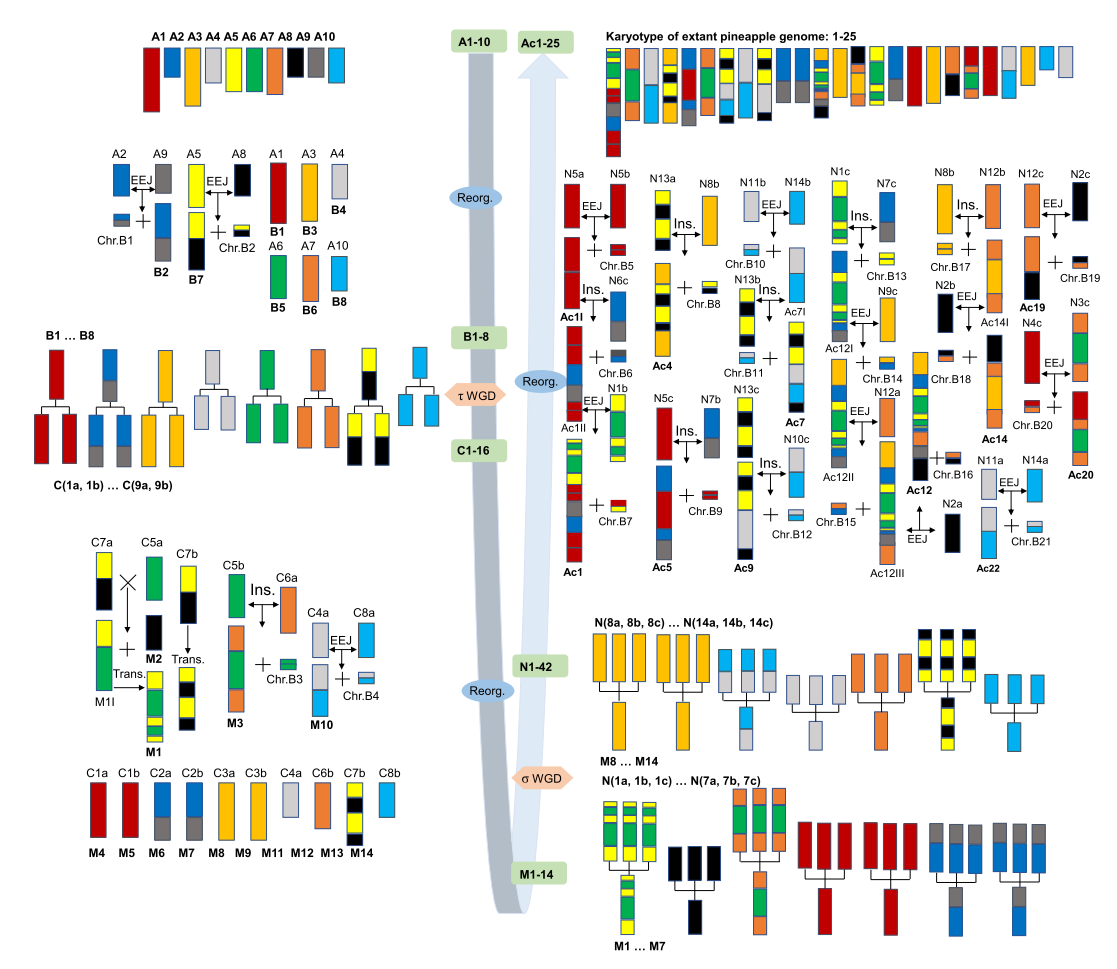


**Fig S15. The inferred evolutionary trajectories to form extant *Ananas comosus* chromosomes.**

The sign of “EEJ” represents “end end join”; the sign of “Reorg.” represents reorganization; the sign “Ins.” represents insertion. the sign of “Chr. B” represents mini-chromosome (B chromosome) produced by the “EEJ” process.


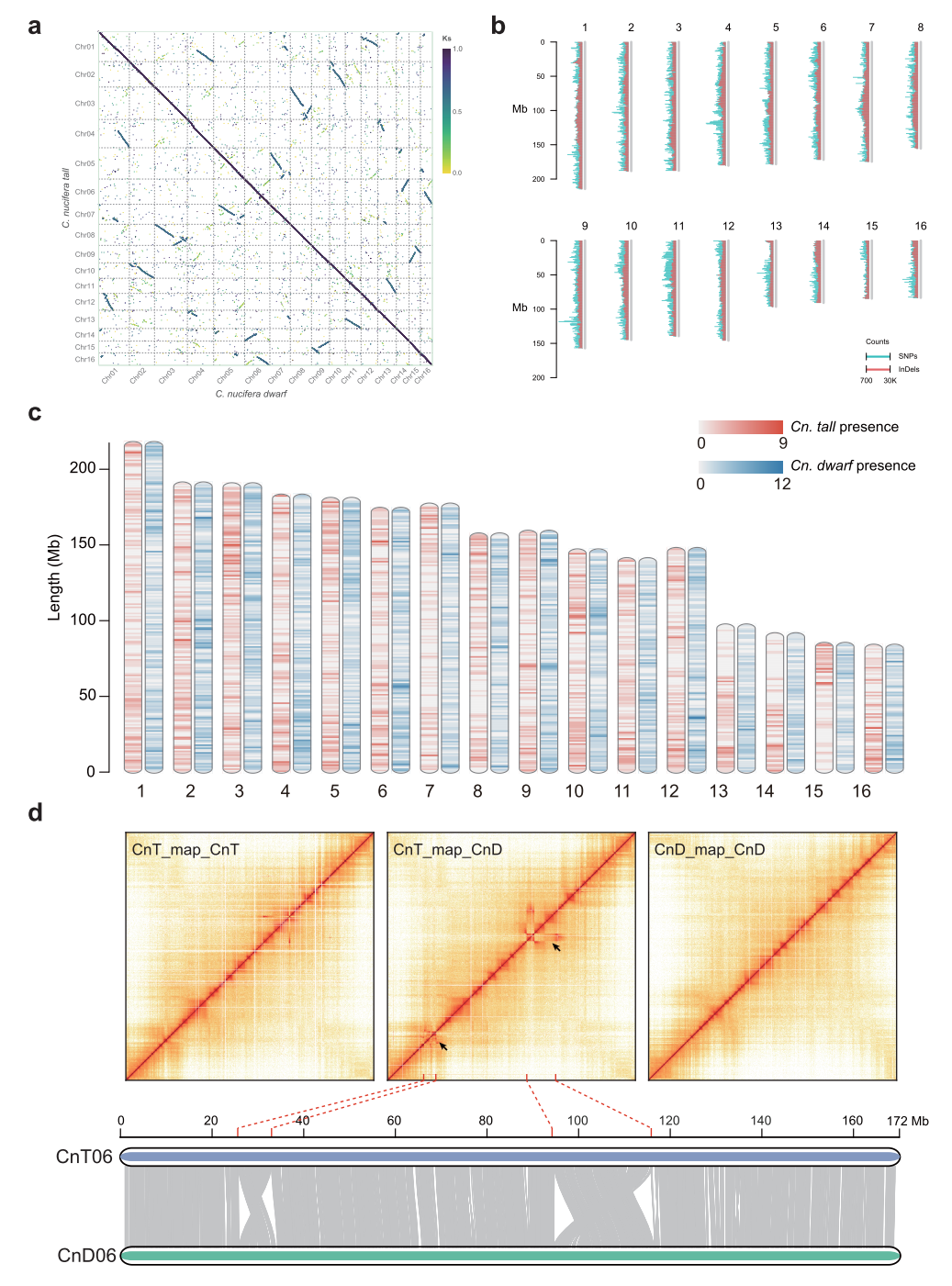


**Fig S16. Genome variation between *Cn. tall* and *Cn. dwarf*.**

**a**, Collinear blocks and Ks value between *Cn. tall* and *Cn. dwarf*. **b**, The distribution of SNPs/InDels on coconut chromosomes. *Cn. tall* is showed as reference genome. **c**, The distribution of PAVs on chromosomes. **d**, Identification of large structural variation in Chr06. CnT_map_CnT indicates mapping *Cn. tall* Hi-C data to *Cn. tall* genome, CnT_map_CnD indicates mapping *Cn. tall* Hi-C data to *Cn. dwarf* genome, CnD_map_CnD indicates mapping *Cn. dwarf* Hi-C data to *Cn. dwarf* genome.


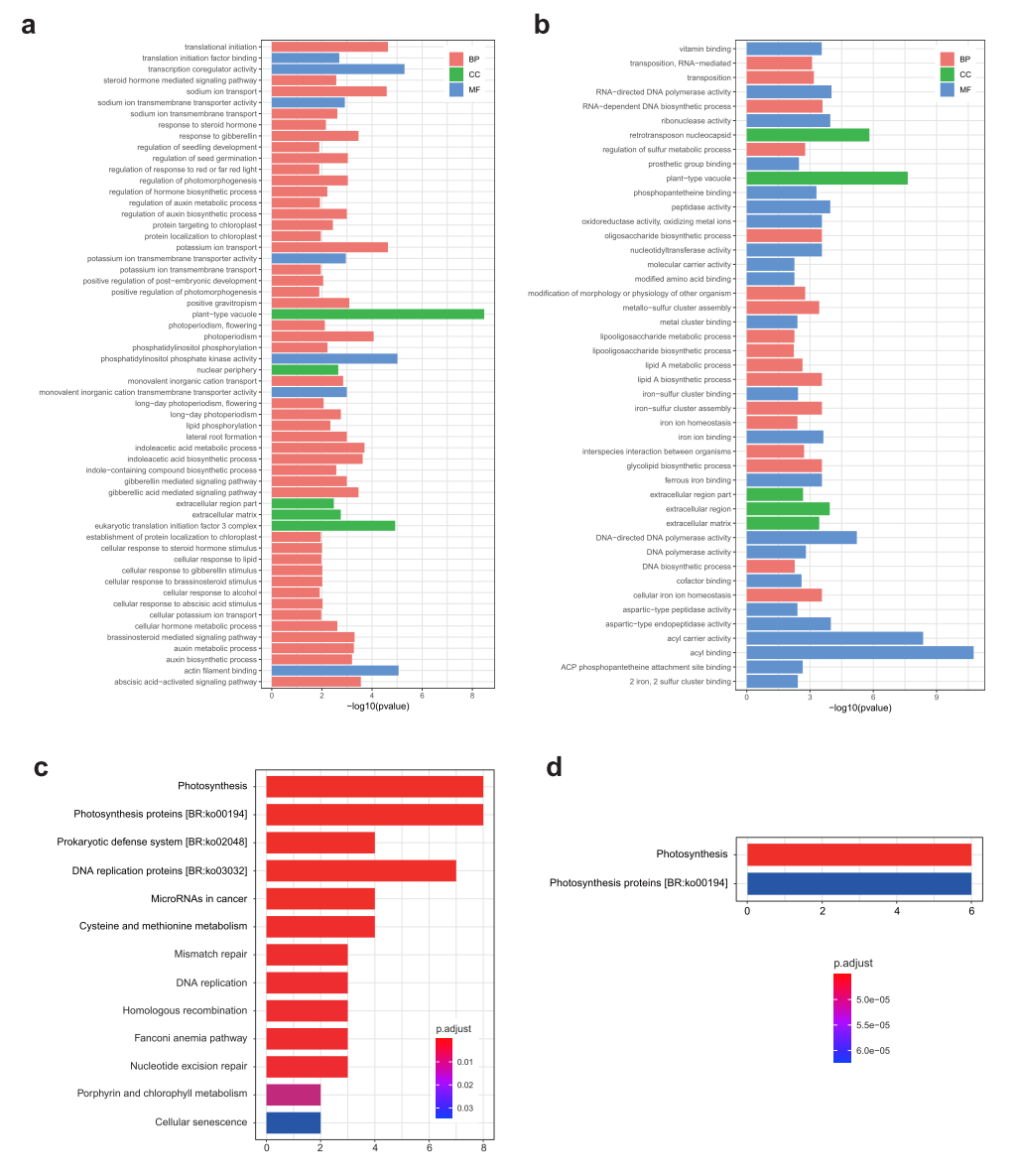


**Fig S17. Enrichment analysis of specific genes.**

**a**, GO enrichment analysis of *Cn. tall*-specific genes. **b**, KEGG enrichment analysis of *Cn. tall*-specific genes. **c**, GO enrichment analysis of *Cn. dwarf*-specific genes. **d**, KEGG enrichment analysis of *Cn. dwarf*-specific genes.


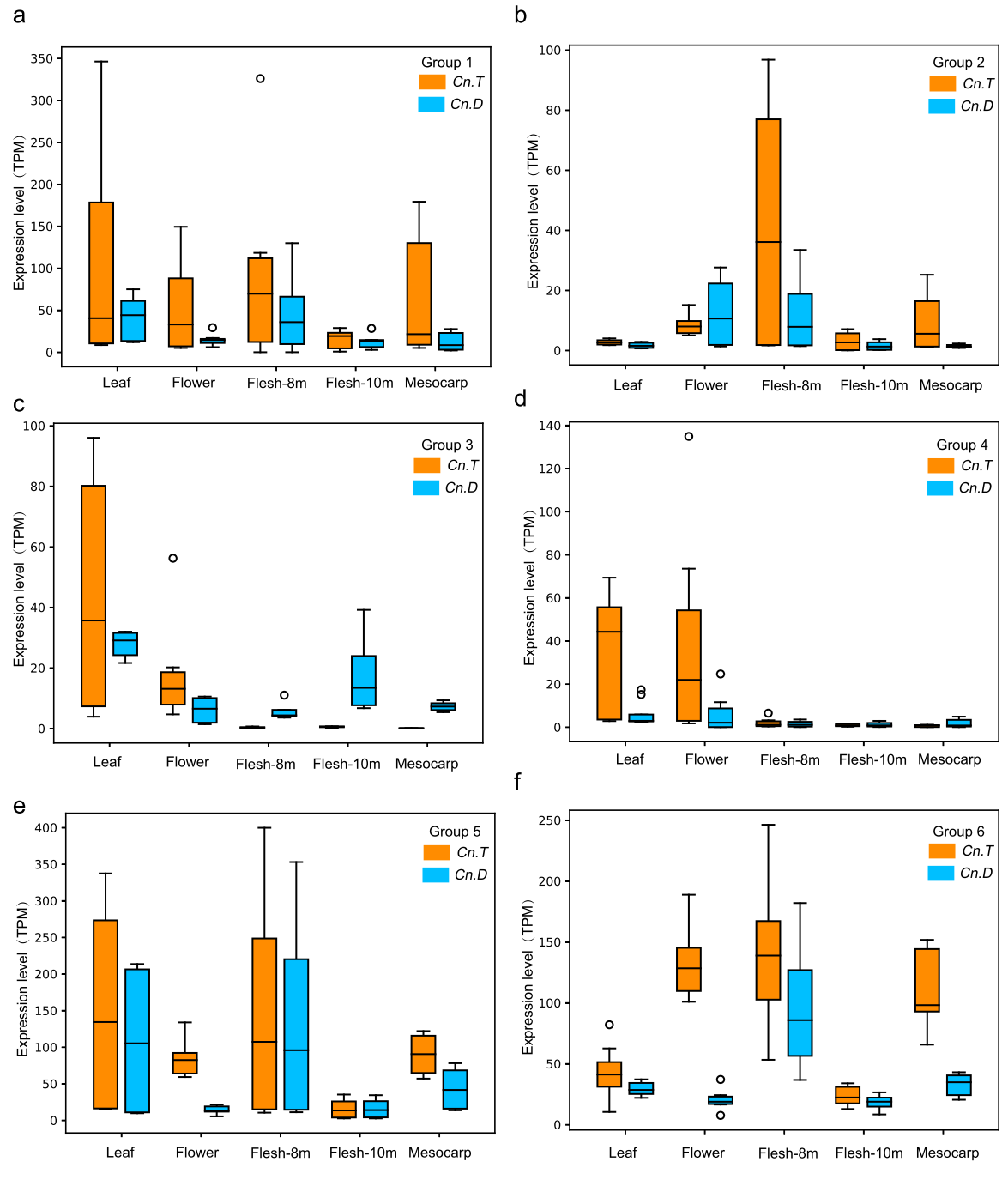


**Fig S18. The expression pattern of CESA gene in different tissues of coconut.**

**a-f**, The expression level of CESA gene of each group in different coconut tissues.


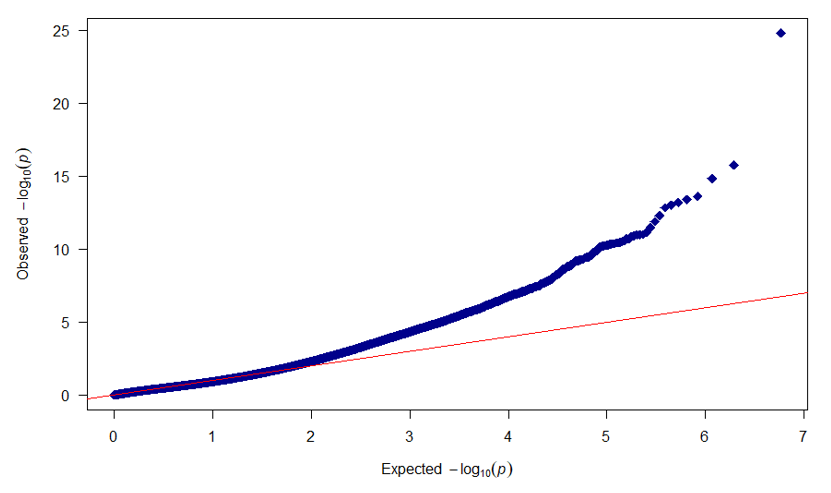


**Fig S19. Quantile-quantile plot for height phenotype.**

Quantile-quantile plot for plant height in coconut populations.


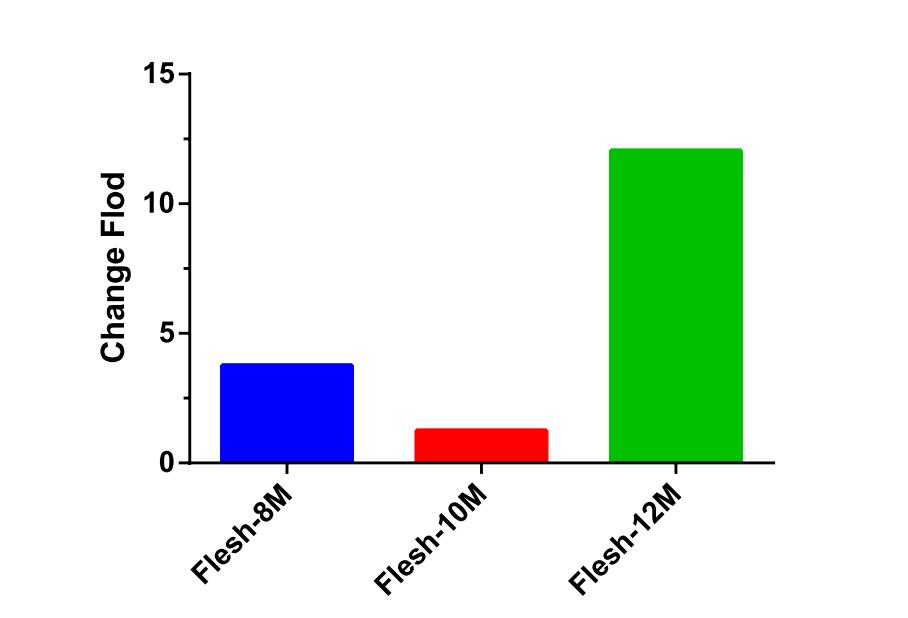


**Fig S20. Statistics on change fold of GA20ox expression on Chr. 12 with *Cn.tall* compare *Cn.dwarf***.
