# Supplementary material for: High-quality reference genome sequences of two coconut cultivars provide insights into evolution of monocot chromosomes and differentiation of fiber content and plant height
Source: Genome Biol. 2021 Nov 4;22:304. doi: 10.1186/s13059-021-02522-9 (PMC8567702; doi:10.1186/s13059-021-02522-9)
Supplement: Supplementary file 16 — Additional file 16: Note S1. More information about inferring ancient monocot karyotypes and evolutionary trajectories to form extant chromosomes. [file 13059_2021_2522_MOESM16_ESM.docx]

Supplementary Note

**Note S1. More information about inferring ancient monocot karyotypes and evolutionary trajectories to form extant chromosomes.**

In a hierarchical manner, we deduced the ancestral chromosomes at the other evolutionary nodes of monocots and their corresponding evolutionary trajectories, respectively. From the node A to *S. polyrrhiza*, the duplicates of proto-chromosomes A4 and A10 merged into a single chromosome, which produced Sp3, Sp8, Sp13, and Sp18, that is, the reorganization of A4 and A10 occurred before the polyploidization β (Additional file 1: Figure S14). Two chromosomal reorganizations occurred during the period between two polyploidizations affecting the lineage to *S. polyrrhiza*, and the proto-chromosomes A5 and A6 were merged into one intermediate chromosome, to produce the full extant chromosome Sp4 and the majority part of chromosome Sp6; while A2 and A9 merged into one intermediate chromosome, to produce part of chromosome Sp1 and the full extant chromosome Sp11. A series of 13 reorganizations after β event occurred, similar to those described above, and finally formed the extant 20 chromosomes of *S. polyrrhiza* (Additional file 1: Figure S14).

*A. comosus* and *C. nucifera* shared the evolutionary trajectory from node A and node C (Fig. 2b). The proto-chromosomes A2 and A9 merged into a chromosome, named B2 at node B, and this was inferred based on the facts that the shared gene collinearity between extant chromosomes Ac1, Ac11, Ac16, Ac5, Ac10 and Ac12 (Additional file 1: Figure S13b and 15), among which the former three chromosomes and the latter three ones were obviously triplicates produced from M6 (C2a) and M7(C2b) by sigma (σ) WGT, respectively. While C2a and C2b were produced by the duplicated of B2, which was produced by joining A2 to A9 (Additional file 1: Figure S15). Likely, the merge of A5 and A8 at node B before tau (τ) WGD could be evidenced by collinear blocks involving chromosomes Ac1, Ac4, Ac7, Ac9 and Ac12, to *S. polyrrhiza* chromosomes (Additional file 1: Figure S13b and 15).

After tau (τ) WGD, Arecales family (e.g., *C. nucifera)* were split from Poales and the other Commelinid families and we inferred chromosome evolutionary trajectories, with *A. comosus* representing Poales and *C. nucifera* representing Arecales. For Poales branch, from node C to *A. comosus*, we deduced that the chromosome rearrangements during the period from tau (τ) WGD to sigma (σ) WGT (Additional file 1: Figure S15). The combination of C7a and C5a (represented by Sp4 and Sp6) could be evidenced by gene collinearity between Sp4/Sp6 and Ac15/Ac1, which were two duplicates produced by the sigma (σ) WGT, but not associated with tau (τ) WGD. We could infer this reorganization pattern to have appeared during the period from tau (τ) WGD to sigma (σ) WGT. The extant chromosomes of Ac2, Ac6 and Ac20 were produced by sigma(σ) WGT after the insertion of C5a into C5b, forming chromosome M3 at node M (Additional file 1: Figure S15). This could be reflected by the corresponding homologous dotplot (Additional file 1: Figure S13b). We have to clarify, the insertion could be inferred by the shared gene collinearity of Ac2, Ac6 and Ac20, while the chromosome Ac12 was formed by joining two ancestral chromosomes, proving that the reorganization only occurred during the period from tau (τ) WGD to sigma (σ) WGT. By joining C8a and C4a to form M10, the intermediate chromosome was tripled into chromosome Ac3, Ac8, and middle part of Ac9 after sigma (σ) WGT. Ac22 was produced by joining C4b and C8b, while Ac24 and Ac25 were derived from C4b and C8b without any recombination, respectively (Additional file 1: Figure S15).

As to the Arecales branch, by comparing with the ancestral karyotype represented by *S. polyrrhiza* chromosomes (Additional file 1: Figure S13a), we inferred that the Arecales ancestral plant had subjected to relatively complex karyotype changes to form *C. nucifera* chromosomes (Fig. 2c). By analyzing gene collinearity between *S. polyrrhiza* and *C. nucifera*, seven chromosomal reorganizations were inferred to have occurred during the period from tau (τ) WGD to sigma (σ) WGT. C7a (produced by joining A5 and A8) and C2a (produced by joining A2 and A9) merged into ancestral chromosome D1 at node D, which eventually evolved into Cn3 and Cn8 after the ω WGD within Arecaceae family (Fig. 2b). C4a (derived from A4) and C8a (derived from A10) merged to produce D8 and D9, respectively (Fig. 2b, c). C6a and C6b (duplicated from A7) merged into an intermediate chromosome, which then was inserted of C1a (derived from A1) into D4. Cn7 and part of Cn5 (duplicates by D4 after ω WGD) were derived from their ancestral chromosomes. The complex formation of D2 and D3, at node D, were associated with four chromosomes, C5a, C7b, C5b and C2b. An insertion of C5a, into C7b produced two intermediate chromosomes, both of which suffered another time of chromosomal arms exchange between C5b and C2b, respectively (Fig. 2b, c).

As to complex structures of the other studied monocots (*M. acuminate*, *A. officinalis* and *O. sativa*), their putative karyotypes were directly reconstructed by exploring the gene collinearity with the ancestral chromosomes, which were represented by *S. polyrrhiza* genome (Fig. 2a, Additional file 1: Figure S13d-f), respectively, and their evolutionary trajectories were not inferring here.
