# Supplementary material for: High-quality reference genome sequences of two coconut cultivars provide insights into evolution of monocot chromosomes and differentiation of fiber content and plant height
Source: Genome Biol. 2021 Nov 4;22:304. doi: 10.1186/s13059-021-02522-9 (PMC8567702; doi:10.1186/s13059-021-02522-9)
Supplement: Supplementary file 24 — Additional file 24. Review history. [file 13059_2021_2522_MOESM24_ESM.docx]

Review History

**First round of review**

**Reviewer 1**

**Are you able to assess all statistics in the manuscript, including the appropriateness of statistical tests used?**

Yes, and I have assessed the statistics in my report.

**Comments to author:**

Coconut is an important oil and fruit crop. It occupies an important phylogenetic position for investigating the evolutionary history of monocots. Wang et al. described high-quality genome sequences of the two basic coconut varieties, Cocos nucifera tall and Cocos nucifera dwarf. The authors performed comparative genomics analysis between the two genomes and identified an Arecaceae-specific WGD event. They also reconstructed a putative karyotype of the ancestor genome of monocots and proposed evolutionary trajectories of coconut and several other monocot genomes. This manuscript represents good genomic work that delivered some interesting results such as chromosomal evolution of monocots. The manuscript is valuable for coconut improvement through genome-assisted breeding as well as plant evolutionary studies. Overall, the work presented in this manuscript is novel and impactful, and it should be published after some minor details are resolved.

Pg 2 Line 38, Line 57, Pg 4, Line 104, "Aracaceae" should be "Arecaceae".

Pg 9, Line 242, " the ancestor of C. nucifera and E. guineensis had 10 proto-chromosomes (Fig. 2b)". Should it be 9 proto-chromosomes according to Fig. 2b?

Pg 9, Line 245 - 248, "Through two independent end-end join (EEJ), …, with A2 and A5 joining to produce B2, and A9 and A8 joining to produce B7,…" According to Fig 3, it should be "A2 and A9 joining to produce B2, and A5 and A8 joining to produce B7".

Pg 10, Line 287-290, Based on the result shown in Fig. 4d, the expression level of the serine carboxypeptidase gene (GZ15G0286110) is very low. It's unlikely that the SV of this region causes the phenotypic difference in cellulose content between Cn. tall and Cn. dwarf.

Pg 10, Line 276, "The variables TEs" should be "The variable TEs".

The GenBank accession number of the RNA-Seq data of Cn. dwarf couldn't be found in Table S4.

Pg 9, Line 266, "among which 37,672 were absent in Cn. tall and 31,340 segments in Cn. dwarf". Are these 31,340 segments absent or present in Cn. dwarf? Please specify.

Pg9, Line 269, "…showed that they were significantly differentially enriched…" "Differentially" needs to be removed.

In the figure legend of Figure S12 C, KEGG should be GO?

Pg 12, Line 331-333, "the expression of transcription factors A, B, C that regulate … displayed similar expression patterns (Fig. 5e)." What are the transcription factors A, B, C? Please specify. According to the Fig. 5e, they don't really show similar expression patterns.

Are the expression patterns of CESA genes shown in Fig, 5g the total expression of all the CESA genes identified in the coconut genome? There are more CESA genes of P3 and P4 clades in Cn. tall than in Cn. dwarf . Why not compare the expression patterns of CESA genes in P3 and P4 clades?

Pg 13, Lin 370-378, authors identified the SNP associated with internode distance and also found copy number variations of GA20ox between Cn. tall and Cn. dwarf ? How about the expression patterns of these genes in Cn. tall and Cn. dwarf ?

Pg 16, Line 473, "The results presented her …" "her" should be "here".

Pg 17, Line 484, for the same reason I described above, it didn't convince me that the SV on the Chr. 15 caused the phenotypic difference in cellulose content between Cn. tall and Cn. dwarf.

Pg 17, Line 485, " mainly genes related to lignin and cellulose…" "mainly" should be "main".

In the figure legend of Fig. 4c, "LTRs" needs to be changes to "TEs"?

In the figure legend of Fig. 4d, what are CH, JJ, XH, YR?

Pg 32, Line 931, "are indicated with orange, green and other gene". "other gene" needs to be changes to "blue".

In the figure legend of Fig. 4e, please explain what are highlighted with red color.

The figure legend of Fig. 5c needs more detailed information. What are the numbers?

In the figure legend of Fig. 5f, please explain the naming system of these CESA genes.

**Reviewer 2**

**Are you able to assess all statistics in the manuscript, including the appropriateness of statistical tests used?**

There are no statistics in the manuscript.

**Comments to author:**

Wang et al. contributed their study of coconut genomics. This manuscript brings high-quality genome assemblies, provides insights into monocot genome evolution by ancestral genome reconstruction, and reports the identification of key genes/variants associated with key trait, like tree height, with GWAS and multi-omics tech. In general, the manuscript is well designed and the analyses are comprehensive. Specifically, they focus the variation of plant height by taking integrated multi-omics analysis and revealed that the difference in coconut plant height may come from the altered gibberellin metabolism, with both the GA20ox copy number and a single nucleotide change in the promoter. I suggest to accept this manuscript after making major revisions.

Major comments:

1. Ancestral genome reconstruction and further inference of proto-chromosomes evolution are key topics in the paper, but they are totally lost in M&M.

2. The authors cannot infer the WGD occurred in the common ancestor of Arecaceae simply based on the phylogenetic tree and the Ks. It is worth comparing Daemonorops jenkinsiana, Calamus simplicifollus, Phoenix dactylifera with Cocos nucifera for comparative genomic analysis and analyzing KS in paralogs in syntenic region in these species to provide more evidence that the WGD occurred in the common ancestor of Arecaceae.

3. Line 122：In genome assembly and annotation part. You mentioned that the N50 of the two coconut genomes are 2.9mb and 14.3mb respectively. Since your experimental methods are exactly the same, it is necessary to discuss the large gap of N50. Due to the limitations of nanopore's sequencing technology, it is recommended to check whether there are more continuous repeats in one of the genomes.

4. Lines 395-398: "Based on the above results, we found that coconut plant height is mainly affected by the structural variation of GA20ox and evidences such as transcriptome, metabolome and cytology have further confirmed the difference mechanism of coconut plant height". The problem is that the metabolome and cytology evidence came from the leaves rather than from the stems, it is necessary to make connections in the Discussion.

5. The conclusion should be altered to add the significance of molecular mechanism of plant height in the application of agriculture and forestry.

Minor ones:

1. lines 105-106: the sentence "The additionally ..." is problematic.

2. line 108: better to replace GA20ox with the gibberellin (GA) biosynthetic enzymes GA 20-oxidases (GA20ox)

3. line 122: Same sequencing tech, sequencing depth, and pipeline were used for genome assembly of these two genomes, but we see truly different continuity, as it is reflected in the length of N50. The authors should explain this in detail.

4. line 136: replace "genome sequences" with "genome assemblies".

5. Line 158: the title "Speciation and genome evolution" should be changed, since the word of " Speciation" is not inapposite for the following paragraphs.

6. line 159, "oldest species" is not a good expression. It would be "the most basal lineage of angiosperms".

7. lines 159-163, as the divergence between coconut and Amborella is significant, it is not proper to infer ancient WGD with the comparison of these two species.

8. lines 303-304: The sentence is inappropriate since NAC is encoded by a large gene family, it is impossible only 11 NACs exist in the genome of Cn. tall and only a few NAC exist in other genomes.

9. lines 342-344: Although the homologous genes from cluster P4 don't exist in Arabidopsis, it cannot simply infer that they may be involved in the metabolism and synthesis of palm cellulose. Since only homologous genes from two species were used here, more sequences from other species can change the topology of the tree (the homologous gene of P4 may exist in other species).

**Reviewer 3**

**Are you able to assess all statistics in the manuscript, including the appropriateness of statistical tests used?**

No, I do not feel adequately qualified to assess the statistics.

**Comments to author:**

Coconut is a very important branch of monocot, and has obvious characteristics, much like a tree.

The coconut genome reported in this study has a high level of assembly and is well-annotated. A detailed analysis and excavation were carried out around the characteristics of coconuts.

Although the coconut genome has been reported previously, the quality of its genome is much worse than the coconut genome reported in this article. The main problem is that there are many results of the study, without highlighting the key points, and further organization is needed.

The suggestions are as follows:

1. The Amborella genome is not assembled to the chromosome level, so why not use the water lily genome? It is recommended that part c of fig1 replace amborella with water lily genome.

There are too many figs in fig1. It is recommended to only use a, c, d, and e.

2. It is recommended to use the f part of fig1 as a main fig or a new fig2. It is necessary to show which gene families are coconut-specific, especially those related to coconut-specific characteristics.

3. It is recommended that fig2 and fig3 be merged into one fig, without showing too many details, just show the key points, and a summary or model.

4. The various parts of fig4 are not connected to each other, and the various parts of a figure should be connected or logical, not split. For example, genes involved in d should be reflected in e.

5. The genes involved in fig5 and fig6 should be reflected in fig4 or the new fig2. Rather than having nothing to do with the previous part, it is not even mentioned.

6. The title needs to be revised, without highlighting the highlights of this article. The title Speciation and genome evolution needs to be revised. Speciation is a very specialized vocabulary, and there is no related result in this chapter.

**Authors Response**

**Point-by-point responses to the reviewers’ comments:**

Reviewer #1: Coconut is an important oil and fruit crop. It occupies an important phylogenetic position for investigating the evolutionary history of monocots. Wang et al. described high-quality genome sequences of the two basic coconut varieties, Cocos nucifera tall and Cocos nucifera dwarf. The authors performed comparative genomics analysis between the two genomes and identified an Arecaceae-specific WGD event. They also reconstructed a putative karyotype of the ancestor genome of monocots and proposed evolutionary trajectories of coconut and several other monocot genomes. This manuscript represents good genomic work that delivered some interesting results such as chromosomal evolution of monocots. The manuscript is valuable for coconut improvement through genome-assisted breeding as well as plant evolutionary studies. Overall, the work presented in this manuscript is novel and impactful, and it should be published after some minor details are resolved.

1. Pg 2 Line 38, Line 57, Pg 4, Line 104, "Aracaceae" should be "Arecaceae".

*Reply: Thanks for your careful check, we have corrected them in the revised manuscript.*

2. Pg 9, Line 242, " the ancestor of C. nucifera and E. guineensis had 10 proto-chromosomes (Fig. 2b)". Should it be 9 proto-chromosomes according to Fig. 2b?

*Reply: Thank you so much for your careful check. We have corrected it in the revised manuscript.*

3. Pg 9, Line 245 - 248, "Through two independent end-end join (EEJ), …, with A2 and A5 joining to produce B2, and A9 and A8 joining to produce B7,…" According to Fig 3, it should be "A2 and A9 joining to produce B2, and A5 and A8 joining to produce B7".

*Reply: Thank you so much for your careful check. we have corrected them in the revised manuscript.*

4. Pg 10, Line 287-290, Based on the result shown in Fig. 4d, the expression level of the serine carboxypeptidase gene (GZ15G0286110) is very low. It's unlikely that the SV of this region causes the phenotypic difference in cellulose content between Cn. tall and Cn. dwarf.

*Reply: Thank you for your comment. We very much agree with you that the expression level of the serine carboxypeptidase gene (GZ15G0286110) is very low, and the SV example involving germination is also almost unrelated to other analyses in this manuscript. Therefore, in the revised manuscript, we describe two other SV examples involving GA and cellulose biosynthesis. The detailed information is as follows: a large segment insertion (0.18 Mb) on the chromosome 12 of the Cn. tall genome containing eight genes, one of which was annotated as gibberellin-regulated protein gene (GZ12G0246340) and had higher expression levels in coconut flower and mesocarp than in other tissues, which may have an important impact on coconut plant height traits. Another example involves in a deletion (0.12 Mb) on chromosome 4 of Cn. dwarf resulting in the loss of a cellulose synthase A gene (GZ04G0103690) associated with cellulose biosynthesis, which had very high expression level in coconut mesocarp consisted of cellulose (Fig. 3d).*

5. Pg 10, Line 276, "The variables TEs" should be "The variable TEs". The GenBank accession number of the RNA-Seq data of Cn. dwarf couldn't be found in Table S4.

*Reply: Thanks for your careful check. we have corrected it in the revised manuscript. In addition, we have uploaded the genome and transcriptome related data, and provided the GenBank accession number in Table S4.*

6. Pg 9, Line 266, "among which 37,672 were absent in Cn. tall and 31,340 segments in Cn. dwarf". Are these 31,340 segments absent or present in Cn. dwarf? Please specify.

*Reply: Thank you very much for your comments. These 31,340 segments are absent in the Cn. dwarf. We made it clear in the revised manuscript.*

7. Pg9, Line 269, "…showed that they were significantly differentially enriched…" "Differentially" needs to be removed. In the figure legend of Figure S12 C, KEGG should be GO?

*Reply: Thank you very much for your comments. We corrected them in the revised manuscript.*

8. Pg 12, Line 331-333, "the expression of transcription factors A, B, C that regulate … displayed similar expression patterns (Fig. 5e)." What are the transcription factors A, B, C? Please specify. According to the Fig. 5e, they don't really show similar expression patterns.

*Reply: Thank you very much for your comments. We have revised the results of A, B and C, which are MYB85, MYB58 and MYB46. The expression levels of the transcription factors MYB85, MYB58 and MYB46, which have been reported to regulate the metabolism and synthesis of lignin, were significantly different in various coconut tissues. Compared with Cn. dwarf, almost all of the three MYB transcription factors showed higher expression levels in Cn. tall, especially in the mesocarp. Among them, MYB46 has the highest expression level, indicating that it may play a more important role in the difference in lignin content between the two coconut varieties.*

9. Are the expression patterns of CESA genes shown in Fig, 5g the total expression of all the CESA genes identified in the coconut genome? There are more CESA genes of P3 and P4 clades in Cn. tall than in Cn. dwarf . Why not compare the expression patterns of CESA genes in P3 and P4 clades?

*Rely: Thanks for your comments and suggestions. We have compared the expression of all CESA genes in coconut. Compared with the previous phylogenetic analysis using CESA conservative sequences, we used all non-redundant CESA protein sequences in Arabidopsis and coconut for phylogenetic analysis. In addition, we have added the expression levels of the CESA gene in each group in the Cn. tall and Cn. dwarf in the revised Figure S18. It is observed that the expression level of Cn. tall is significantly higher than that of Cn. dwarf in different tissues.*

10. Pg 13, Lin 370-378, authors identified the SNP associated with internode distance and also found copy number variations of GA20ox between Cn. tall and Cn. dwarf ? How about the expression patterns of these genes in Cn. tall and Cn. dwarf ?

*Reply: Thanks for your comments. In the revised manuscript, we compared the expression levels of these GA20ox in different tissues of Cn. tall and Cn. dwarf. The transcriptome analysis showed that the expression level of GA20ox gene on chromosome 12 of Cn. tall was higher than that of Cn. dwarf, and its fold change ranges from 1.3 to 12 (Additional file 2: Figure S20). In addition, we compared the GA20ox expression level between Cn. tall and Cn. dwarf by qRT-PCR, and the results showed that there was a significant difference of GA20ox expression between the two coconut genomes (Fig. 5d).*

11. Pg 16, Line 473, "The results presented her …" "her" should be "here".

*Reply: Thank you so much for your careful check. we have corrected it in the revised manuscript.*

12. Pg 17, Line 484, for the same reason I described above, it didn't convince me that the SV on the Chr. 15 caused the phenotypic difference in cellulose content between Cn. tall and Cn. dwarf.

*Reply: Thank you for your comment. In the revised manuscript, we re-analyzed structural variation and described two SV examples involving GA and cellulose biosynthesis.*

13. Pg 17, Line 485, " mainly genes related to lignin and cellulose…" "mainly" should be "main".

In the figure legend of Fig. 4c, "LTRs" needs to be changes to "TEs"?

In the figure legend of Fig. 4d, what are CH, JJ, XH, YR?

*Reply: Thank you so much for your careful check. We have revised the contents of line 485 and Fig. 3c (The original figs 2 and 3 have been merged) according to your suggestions. In addition, the legend in Fig. 3d has been updated, and the four different tissues CH, JJ, XH, YR represent leaf, flower, fruit, and mesocarp, respectively. Fruit-10m means fruit-10 months.*

14. Pg 32, Line 931, "are indicated with orange, green and other gene". "other gene" needs to be changes to "blue".

*Reply: Thank you so much for your careful check. We have corrected it in the revised manuscript.*

15. In the figure legend of Fig. 4e, please explain what are highlighted with red color.

*Reply: The red numbers indicate differences in the number of genes between coconuts and other species, we have revised it in the manuscript.*

16. The figure legend of Fig. 5c needs more detailed information. What are the numbers?

*Reply: The red numbers indicate the same number of these genes in Cn. tall and Cn. dwarf, while the purple numbers indicate different numbers. We have revised it in the manuscript.*

17. In the figure legend of Fig. 5f, please explain the naming system of these CESA genes.

*Reply: We obtained the CESA genes of coconut through gene annotation, and numbered these genes in coconut according to the order in which they appeared on chromosomes 1 to 16.*

Reviewer #2:

Wang et al. contributed their study of coconut genomics. This manuscript brings high-quality genome assemblies, provides insights into monocot genome evolution by ancestral genome reconstruction, and reports the identification of key genes/variants associated with key trait, like tree height, with GWAS and multi-omics tech. In general, the manuscript is well designed and the analyses are comprehensive. Specifically, they focus the variation of plant height by taking integrated multi-omics analysis and revealed that the difference in coconut plant height may come from the altered gibberellin metabolism, with both the GA20ox copy number and a single nucleotide change in the promoter. I suggest to accept this manuscript after making major revisions.

Major comments:

1. Ancestral genome reconstruction and further inference of proto-chromosomes evolution are key topics in the paper, but they are totally lost in M&M.

*Reply: Thanks for your comments and suggestions. The deduction of karyotype of ancient genome and the trajectory of extant species’ formation were mainly based on their collinearity between the compared genomes. As to your comment, we have now moved part of the inference description into the Methods part of the main text.*

*Inferring ancestral cell karyotypes and reconstruct chromosome evolutionary trajectories*

*By referring to gene collinearity between compared species, we found that S. polyrrhiza (Sp) and A. comosus (Ac) provided great opportunity to infer ancestral karyotypes of basal monocots, due mainly to their well-preserved gene collinearity. For example, gene collinearity showed prominent homology between the middle part of Sp chromosome 1 (Sp1), the middle part of Sp6, the former part of Sp7, and the former part of Sp12, which were surely produced by two rounds of polyploidizations (α and β) (Additional file 2: Figure S9c); these listed regions in Sp chromosomes further corresponded to the middle and latter part of Ac chromosome 1 (Ac1), the middle part of Ac5, nearly whole chromosome Ac17 and chromosome Ac21, and the majority of Ac20. The paralogs between these Ac chromosomes were produced by sigma (σ) whole-genome triplication (WGT) (Fig. 2b, Additional file 2: Figure S11), which are shared across the Poales plants. Actually, the above chromosome homology supported that they were derived from ancestral chromosomes M4 and M5 (Fig. 2a, Additional file 2: Figure S11), which were inferred to have been produced by duplicating the proto-chromosome A1 since the node A before the split with S. polyrrhiza. By characterizing gene collinearity between S. polyrrhiza and C. nucifera, A. officinalis, and V. vinifera, respectively, the evolutionary trajectory of proto-chromosome A1 obtained further support. The evolutonary trajectory of proto-chromosome A3 was deduced by analyzing gene collinearity of S. polyrrhiza (Sp) and A. comosus (Ac), from which we found that the latter part of Sp2, Sp3 and the latter part of Sp7 (produced by α and β) corresponded to one group of chromosomes (Ac4, Ac13 and Ac14) and the other one group (Ac12, Ac18 and Ac23). Besides, several chromosomes were directly inherited from the proto-chromosomes. For example, the extant chromosomes Sp15, Sp13, Sp16, Sp20, Ac24, and Ac25 were derived directly from proto-chromosomes A6, A7, A8, A9, A10, and A4, respectively. While the evolutionary trajectories of A2 and A5 were more complex, and each of its corresponding duplicated chromosomes was reorganized with the other chromosomes derived from proto-chromosomes (Additional file 2: Figure S11).*

*In a hierarchical manner, we deduced the ancestral chromosomes at the other evolutionary nodes of monocots and their corresponding evolutionary trajectories, respectively. See details in Additional file 3.*

2. The authors cannot infer the WGD occurred in the common ancestor of Arecaceae simply based on the phylogenetic tree and the Ks. It is worth comparing Daemonorops jenkinsiana, Calamus simplicifollus, Phoenix dactylifera with Cocos nucifera for comparative genomic analysis and analyzing KS in paralogs in syntenic region in these species to provide more evidence that the WGD occurred in the common ancestor of Arecaceae.

*Reply: Thanks for your comments and suggestions. Through comparing collinearity of the other four recently released Arecaceae species with coconut, we found that all the compared Arecaceae went through two rounds of WGDs (Additional file 2: Figure S7), within which one recent polyploidization (ω WGD) shared with coconut and likely all Arecaceae family and another more ancient one was τ WGD (Additional file 2: Figure S8). A detailed analysis separated the best-matched chromosomes or chromosome regions, being orthologous between genomes, and secondly matched ones, produced by shared WGDs by different plants. As an example, we found that Areca catechu chromosome 5 (Ac5) is best-matched with Ac3 and secondarily matched with overlapping regions in Ac9 and Ac12 (Additional file 2: Figure S7a), and compared with coconut (Cn13), it was best-matched with Ac3, secondarily matched with Ac5, and regions in Ac9 and Ac12 (Additional file 2: Figure S8a). Ks distribution analysis of collinear genes within and between Arecaceae genomes (Additional file 2: Figure S9 a-f) showed two peaks in each genome, with the first Ks appearing at about 0.30-0.42 and the other one appearing at about 0.89-0.96 (Additional file 2: Figure S9g, Additional file 1: Table S12), also supporting the shared WGDs in Arecaceae plants.*

3. Line 122：In genome assembly and annotation part. You mentioned that the N50 of the two coconut genomes are 2.9mb and 14.3mb respectively. Since your experimental methods are exactly the same, it is necessary to discuss the large gap of N50. Due to the limitations of nanopore's sequencing technology, it is recommended to check whether there are more continuous repeats in one of the genomes.

*Reply: Thanks for your comments and suggestions. The Cn. tall genome was sequenced with Nanopore GridION in 2019, while Cn. dwarf genome was sequenced with Nanopore PromethION in 2020. Although the constructed sequencing libraries of the two genomes were similar, the sequencing read length distribution of the two genomes was different. In the following Table 1, it is observed that the mean read length of Cn. dwarf (19.47 kb) is much higher than that of Cn. tall (14.86 kb), and the number (or ratio) of long sequencing reads in Cn. dwarf is also much higher than that in Cn. tall genome, therefore, the assembled contig N50 in Cn. dwarf is much higher than that in Cn. tall.*

*Table 1. Statistics of sequencing read length in Cn. dwarf than Cn. tall genome*

*Sequencing data (Gb) Reads >20kb (%) Reads >40kb (%) Mean read length (kb)*

*Cn. tall 234.2 5,328,066 (30.1%) 1,000,398 (5.7%) 14.8*

*Cn. dwarf 201.7 5,166,072 (39.7%) 1,420,951 (10.9%) 19.5*

4. Lines 395-398: "Based on the above results, we found that coconut plant height is mainly affected by the structural variation of GA20ox and evidences such as transcriptome, metabolome and cytology have further confirmed the difference mechanism of coconut plant height". The problem is that the metabolome and cytology evidence came from the leaves rather than from the stems, it is necessary to make connections in the Discussion.

*Reply: We very much agree with your suggestion, and we modified it in the discussion section of the revised manuscript. The mature stem of coconut is a highly fibrotic tissue, which makes the analysis of the transcriptome and metabolome very difficult. Studies have shown that GA20ox is highly expressed in plant tender leaves, unopened flowers and other tissues, and is a key gene for GA metabolism. Therefore, we selected different young coconut tissues to study the molecular mechanism of plant height differences.*

5. The conclusion should be altered to add the significance of molecular mechanism of plant height in the application of agriculture and forestry.

*Reply: We very much agree with your opinion and summarized the significance of modern molecular markers in the application of agricultural and forestry breeding in the conclusion section. Traditional breeding techniques are still difficult for coconuts because of its long lifecycle. We can analyze the genetic basis of key traits such as plant height and fiber content through multi-omics methods, and develop relevant molecular markers based on the genetic variation of their traits. Moreover, modern molecular marker breeding technology will speed up the process of coconut nutrient quality improvement and precise breeding of new varieties.*

Minor ones:

1. lines 105-106: the sentence "The additionally ..." is problematic.

*Reply: OK, we have revised it.*

2. line 108: better to replace GA20ox with the gibberellin (GA) biosynthetic enzymes GA 20-oxidases (GA20ox)

*Reply: OK, we have revised it.*

3. line 122: Same sequencing tech, sequencing depth, and pipeline were used for genome assembly of these two genomes, but we see truly different continuity, as it is reflected in the length of N50. The authors should explain this in detail.

*Reply: Thanks for your comments and suggestions. The Cn. tall genome was sequenced with Nanopore GridION in 2019, while Cn. dwarf genome was sequenced with Nanopore PromethION in 2020. Although the constructed sequencing libraries of the two genomes were similar, the sequencing read length distribution of the two genomes was different. In the above Table 1 in this response, we observed that the mean read length of Cn. dwarf (19.47 kb) is much higher than that of Cn. tall (14.86 kb), and the number (or ratio) of long sequencing reads in Cn. dwarf is also much higher than that in Cn. tall genome, therefore, the assembled contig N50 in Cn. dwarf is much higher than that in Cn. tall.*

4. line 136: replace "genome sequences" with "genome assemblies".

*Reply: OK, we have revised it.*

5. Line 158: the title "Speciation and genome evolution" should be changed, since the word of " Speciation" is not inapposite for the following paragraphs.

*Reply: Thank you for your suggestion. The title "Speciation and genome evolution" had been changed into “Genome duplication and evolution”.*

6. line 159, "oldest species" is not a good expression. It would be "the most basal lineage of angiosperms".

*Reply: OK, we have revised it.*

7. lines 159-163, as the divergence between coconut and Amborella is significant, it is not proper to infer ancient WGD with the comparison of these two species.

*Reply: The sentence “indicating that C. nucifera may have undergone two rounds of whole-genome duplications (WGDs)” had been deleted in revised manuscript.*

8. lines 303-304: The sentence is inappropriate since NAC is encoded by a large gene family, it is impossible only 11 NACs exist in the genome of Cn. tall and only a few NAC exist in other genomes.

*Reply: Yes, the NAC family is indeed a large family. Due to the misuse of the HMM file in the Pfam database, only part of NAC family genes in these species have been identified. We re-identified the NAC family genes in these species, and finally obtained 140, 137, 129, 181 and 97 NAC family genes in Cn. tall, Cn. dwarf, E. guineensis, M. acuminate and O. sativa. The corresponding content has been corrected in the revised manuscript.*

9. lines 342-344: Although the homologous genes from cluster P4 don't exist in Arabidopsis, it cannot simply infer that they may be involved in the metabolism and synthesis of palm cellulose. Since only homologous genes from two species were used here, more sequences from other species can change the topology of the tree (the homologous gene of P4 may exist in other species).

*Reply: This is a problem of incorrect expression, and we have revised the content about the identification of coconut CESA genes. We re-analyzed and drew the phylogenetic tree of CESA, and added a new supplementary figure showing the difference in expression of all CESA in Cn. tall and Cn. dwarf. Through blast analysis with Arabidopsis thaliana, a total of 29 CESA genes were identified in coconuts, which were divided into 6 groups, among which there were 15 and 14 in Cn. tall and Cn. dwarf, respectively. Among them, the coconut CESA sequence of group 1 was significantly different from that of Arabidopsis, but it does not mean that it is a specific CESA gene in coconut. Transcriptome analysis showed that CESA expression in Cn. tall was generally higher than gene expression in Cn. dwarf, which is one of the important factors for the fiber content of Cn. tall to be higher than that in Cn. dwarf.*

Reviewer #3:

Coconut is a very important branch of monocot, and has obvious characteristics, much like a tree.

The coconut genome reported in this study has a high level of assembly and is well-annotated. A detailed analysis and excavation were carried out around the characteristics of coconuts.

Although the coconut genome has been reported previously, the quality of its genome is much worse than the coconut genome reported in this article. The main problem is that there are many results of the study, without highlighting the key points, and further organization is needed.

The suggestions are as follows:

1. The Amborella genome is not assembled to the chromosome level, so why not use the water lily genome? It is recommended that part c of fig1 replace amborella with water lily genome.

There are too many figs in fig1. It is recommended to only use a, c, d, and e.

*Reply: Thanks for your comments and suggestions, and we have replaced the Amborella genome with water lily genome in Fig. 1c in the revised manuscript. According to your suggestion, the original Fig. 1b was placed in the Supplementary Figures in the revised manuscript.*

2. It is recommended to use the f part of fig1 as a main fig or a new fig2. It is necessary to show which gene families are coconut-specific, especially those related to coconut-specific characteristics.

*Reply: Thanks for your suggestions. Considering that the content of original Fig. 1f (gene family analysis) is very different from Fig. 2 (Ancestral karyotype of monocots and coconut karyotype evolution), it is still placed in Fig. 1e. The gene family analysis of coconut and other three Arecaaceae species has been re-analyzed, and a supplementary figure of the enrichment analysis of the coconut gene family has been added. We found that 70.32% (10548/15001) of the gene families in C. nucifera were shared among all four genomes, and only 11.86% (1779/15001) of gene families were C. nucifera specific (Fig. 1e). GO enrichment analysis of these gene families revealed 31 significantly enriched GO categorizes, mainly concentrated in biological process category, including ‘vitamin metabolic process’, ‘terpenoid biosynthetic process’ and ‘alpha-amino acid biosynthetic process’ (Additional file 2: Figure S11).*

3. It is recommended that fig2 and fig3 be merged into one fig, without showing too many details, just show the key points, and a summary or model.

*Reply: Thanks for your suggestions. We have merged Fig. 2 and Fig. 3, and further condensed important conclusions in the revised manuscript.*

4. The various parts of fig4 are not connected to each other, and the various parts of a figure should be connected or logical, not split. For example, genes involved in d should be reflected in e.

*Reply: Thank you for your comments and suggestions. In Fig. 3d, we re-analyzed the structural variation and described two SV examples involving GA and cellulose biosynthesis. In Fig. 3e, we analyzed the gene copy number and structure from the four key traits of coconut salt resistance, fiber content, plant height and fatty acid content. At the same time, the molecular mechanisms of fiber content and plant height are analyzed by multi-omics methods in Fig. 4 and Fig. 5. This study focuses on the key traits of coconut analysis to make the logic of the article clearer.*

5. The genes involved in fig5 and fig6 should be reflected in fig4 or the new fig2. Rather than having nothing to do with the previous part, it is not even mentioned.

*Reply: According to your suggestion, we have further highlighted the correlation and logic between Fig. 4 and Fig. 5/6 in the revised manuscript. The genes in Fig. 4e mainly play a role in salt tolerance, fiber content, plant height, and fatty acid content. The difference in the number of above genes may cause differences in the traits of Cn. tall and Cn. dwarf. In Fig. 5 and Fig. 6, we analyzed the molecular mechanism for fiber content difference and plant height between Cn. tall and Cn. dwarf based on high-quality genomes and integrated multi-omics, which are the specific and in-depth research results of Fig. 4.*

6. The title needs to be revised, without highlighting the highlights of this article. The title Speciation and genome evolution needs to be revised. Speciation is a very specialized vocabulary, and there is no related result in this chapter.

*Reply: We have rewritten the title of this article and revised the subtitle “Speciation and Genome Evolution” into “Genome Duplication and Evolution”.*

**Second round of review**

**Reviewer 1**

The authors have addressed my questions and concerns. The revised manuscript can be accepted for publication.

**Reviewer 2**

I noticed the authors have addressed the questions I put forwarded, thus I agree to accept it for publication.

**Reviewer 3**

The author solved my previous questions very well, and I have no question now.
